# Engineering of Reductive Aminases for Asymmetric Synthesis of Enantiopure Rasagiline

**DOI:** 10.3389/fbioe.2021.798147

**Published:** 2021-12-22

**Authors:** Kai Zhang, Yuanzhi He, Jiawei Zhu, Qi Zhang, Luyao Tang, Li Cui, Yan Feng

**Affiliations:** State Key Laboratory of Microbial Metabolism, Joint International Research Laboratory of Metabolic and Developmental Sciences, School of Life Sciences and Biotechnology, Shanghai Jiao Tong University, Shanghai, China

**Keywords:** reductive aminase, chiral amine, site saturation mutagenesis, rational design, rasagiline

## Abstract

Reductive aminases (RedAms) for the stereoselective amination of ketones represent an environmentally benign and economically viable alternative to transition metal–catalyzed asymmetric chemical synthesis. Here, we report two RedAms from *Aspergillus calidoustus* (*Ac*RedAm) and bacteria (*Ba*RedAm) with NADPH-dependent features. The enzymes can synthesize a set of secondary amines using a broad range of ketone and amine substrates with up to 97% conversion. To synthesize the pharmaceutical ingredient (*R*)-rasagiline, we engineered *Ac*RedAm through rational design to obtain highly stereoselective mutants. The best mutant Q237A from *Ac*RedAm could synthesize (*R*)-rasagiline with >99% enantiomeric excess with moderate conversion. The features of *Ac*RedAm and *Ba*RedAm highlight their potential for further study and expand the biocatalytic toolbox for industrial applications.

## Introduction

Chiral amines are valuable building blocks of numerous natural products, active pharmaceuticals, and other high-value chemicals (Afanasyev et al., 2019; Newman and Cragg, 2020; Guo et al., 2021; Li et al., 2021; Nguyen and Kou, 2021). Constituting approximately 40% of new drugs approved by the FDA in recent years, chiral amines constituted a market value of USD 14 billion in 2020 (de Gonzalo and Lavandera, 2021). Numerous chemical strategies have been established for the preparation of chiral amines, which suffer limitations such as low efficiency, low selectivity, and adverse environmental impact. In contrast, enzymes from renewable resources can afford excellent stereo- and regioselectivity and catalyze reactions under mild aqueous conditions (Bornscheuer et al., 2012; Matzel et al., 2017; Wu et al., 2021). Because biochemical reactions can take place without using toxic reagents and extensive protection and deprotection steps, enzymes are always employed as catalysts for developing green chemistry; consequently, biosynthesis has received considerable attention (Ran et al., 2008; Wohlgemuth, 2010; Choi et al., 2015).

Over the last 20 years, a significant number of enzymatic routes have been developed for the synthesis of chiral amines, among which are enzymes that catalyze the reductive amination of prochiral ketones into amines: transaminases (TAs), amine dehydrogenases (AmDHs), and imine reductases (IREDs) have attracted considerable interest (Schrittwieser et al., 2015; Sharma et al., 2017; D Patil et al., 2018; Grogan, 2018; Liu et al., 2020; Montgomery et al., 2020). Both TAs and AmDHs are currently limited to the synthesis of primary amines, necessitating subsequent alkylation chemistry for the synthesis of chiral secondary and tertiary amines (Dold et al., 2016; Knaus et al., 2017). In particular, IREDs can catalyze the NAD(P)H-dependent reduction of prochiral imines to chiral amines (Mitsukura et al., 2010) and prefer the reduction of cyclic imine substrates but lead to poor conversion with the amination of prochiral ketones (Huber et al., 2014; Wetzl et al., 2016).

Notably, Turner et al. reported an NADPH-dependent reductive aminase (*Asp*RedAm) from *Aspergillus oryzae* in 2017, which was identified as a subclass of IREDs and could catalyze intermolecular reductive amination of a wider range of ketones and amines with high activity in aqueous media (Aleku et al., 2017). Compared to the multistep chemical routes and other biocatalysts, including TAs and AmDHs, the RedAm approaches show considerable efficiency in producing all types of chiral amines from ketones and amines in a single condensation step with new C-N single bond formation. In particular, *Asp*RedAm enabled the formation of the anti-Parkinson’s agent (*R*)-rasagiline directly from indanone and propargylamine. In some cases, *Asp*RedAm also displayed high reactivity by catalyzing reductive amination with ketone: amine ratios as low as 1:1 (Aleku et al., 2017).

Subsequently, Turner’s group also disclosed new thermotolerant fungal RedAms that could utilize cheap ammonium salts as amine donors to produce primary amines and perform continuous flow biotransformation under mild conditions (Mangas-Sanchez et al., 2020). Taking advantage of an NADPH cofactor regeneration system and a variety of amines as amino donors, the RedAm-catalyzing process of reductive amination maximizes the atom economy, thereby contributing to environmentally sustainable development. These study results showed that RedAms possess prominent industrial advantages, such as broad substrate scope and excellent stereoselectivity for the synthesis of chiral amines. The remarkable features of known RedAms highlight their great potential for application and have already been successfully applied in industry (Schober et al., 2019).

Currently, the development of RedAms is still limited. Herein, we aimed to apply a sequence structure mining strategy to explore new RedAms for enabling the synthesis of some active pharmaceutical ingredients and scaffolds and engineer them for the synthesis of desired enantiopure products combined with directed evolution. The asymmetric synthesis of rasagiline was chosen as the model reaction because (*R*)-rasagiline is an effective anti-Parkinson’s agent, and (*S*)-rasagiline has also been proven to provide prominent cardioprotective activity (Chen et al., 2007; Berdichevski et al., 2010; Leegwater-Kim and Bortan, 2010; Weinreb et al., 2010; Ertracht et al., 2011).

In this study, the enzymatic properties of the candidate RedAms were investigated, they were successfully engineered to produce enantiopure rasagiline, and their synthetic potential as catalysts for accessing rasagiline in one step was explored.

## Material and Methods

### Strains, Plasmids, and Chemicals

Commercially available chemicals and reagents were purchased from Bidepharm (Shanghai, China), Macklin (Shanghai, China), Energy Chemical (Anhui, China), TCI (Shanghai, China), Kai Wei Chemical (Shanghai, China), Aladdin (Shanghai, China), J&K (Beijing, China), Amethyst (Beijing, China), Collins (Shanghai, China), Adamas (Shanghai, China), Sigma-Aldrich (St. Louis, MO, United States), or Acros Organics (Geel, Belgium) unless stated otherwise. Glucose dehydrogenase (GDH) was purchased from Aladdin (Shanghai, China). Restriction enzymes, T4 DNA ligase, and DNA polymerases were purchased from New England Biolabs or Takara Bio. Chemically competent *E. coli* Trans5α and BL21(DE3) were purchased from TransGen Biotech (Beijing, China). pET28a was obtained from our laboratory. The codon-optimized genes for the candidate RedAms were synthesized and cloned into pUC18 using GenScript (Nanjing, China). All kits for mini-preparation of DNA, PCR purification, and gel extraction were purchased from Axygen (Hangzhou, China).

### Phylogenetic Tree Building and Protein Sequence Structure Analysis

Homologous protein sequences were searched using the BLASTP algorithm (Altschul et al., 2005). The previously reported protein sequences of *Asp*RedAm (accession no. XP_001827659) and *At*RedAm (accession no. XP_001217087) were used as search queries in the GenBank non-redundant protein sequence database. Multiple sequence alignments were performed using ClustalW (Thompson et al., 1994). The phylogenetic tree was constructed using MEGA7 with maximum likelihood algorithms (Kumar et al., 2016). The protein sequences in the phylogenetic tree were submitted to SWISS-MODEL for modeling (Waterhouse et al., 2018). The predicted protein structures were aligned using PyMOL to analyze and compare the structural similarities and active pockets with *Asp*RedAm.

### Gene Cloning, Expression, and Protein Purification

The codon-optimized genes encoding the candidate RedAms were incorporated into the vector pET28a between *Nde*l and *Xho*l restriction sites. The constructed plasmids were confirmed by sequencing and then transformed into *Escherichia coli* BL21(DE3) chemically competent cells by heat shock. The transformed *E. coli* cells were cultivated in 500 ml LB medium with 50 μg/ml kanamycin at 37°C with shaking at 220 rpm. At OD_600_ between 0.6 and 0.8, isopropyl-β-D-thiogalactopyranoside (IPTG) was added to a final concentration of 0.5 mM to induce protein expression. Incubation was continued at 18°C and 220 rpm for 12 h. The cells were then harvested by centrifugation and disrupted by ultrasonication in 100 mM Tris–HCl buffer (pH 8.0). Recombinant proteins were purified from the supernatant by Ni-affinity chromatography. Purified proteins were examined by SDS-PAGE. The concentration of proteins was measured based on the absorbance at 280 nm by using a NanoDrop spectrophotometer (ThermoFisher), and the extinction coefficients were determined by the selected sample type and baseline correction.

### RedAm-Catalyzing Biotransformation

The reductive amination reaction was performed in 100 mM Tris–HCl buffer (pH 9.0) containing 1 mg/ml purified RedAm, 0.7 mg/ml GDH (Aladdin), 30 mM D-glucose, 1 mM NADP^+^, 5 mM ketone, an appropriate ratio of amine (in buffer adjusted to pH 9.0), and 2% (v/v) DMSO. The final reaction volume was made up to 500 µL using Tris–HCl buffer. The reaction mixture was incubated at 25°C with shaking at 220 rpm for 24 h. Then 30 µL of 10 M NaOH was added to quench the reaction. The reaction mixture was extracted twice with 500 µL of *tert*-butyl methyl ether. The organic fractions were combined, dried over anhydrous MgSO_4_, and analyzed using HPLC or GC-FID (Aleku et al., 2017).

Large-scale reactions for synthesizing rasagiline were carried out using 1 mg/ml purified wild-type RedAms or variant, 0.7 mg/ml GDH, 100 mM D-glucose, 1 mM NADP^+^, 5 mM indanone, 250 mM propargylamine, and 2% (v/v) DMSO in 100 mM pH 9.0 Tris buffer. The final reaction volume was made up to 50 ml using Tris–HCl buffer. The reaction mixture was incubated at 25°C with shaking at 220 rpm for 180 h. Then 200 μL of the sample was taken at different time points from 2 to 180 h. The sample was quenched by adding 10 µL of 10 M NaOH and extracted twice with 200 µL of *tert*-butyl methyl ether. The organic layers were combined, dried over anhydrous MgSO_4_, and analyzed using HPLC or GC-FID (Mangas-Sanchez et al., 2020).

### Kinetic Assays

To determine the kinetic parameters of *Ac*RedAm and variants for indanone 9, a typical reaction mixture contained 0.002–10 mM of ketone 9, 20 mM propargylamine e from buffer stock adjusted to pH 9.0, 0.2 mM NADPH, 1% DMSO, and purified enzymes at appropriate concentrations in a total volume of 200 µL (100 mM Tris-HCl, pH 9.0). Activity measurements were performed in triplicate at 340 nm (ε = 6.22 mM^−1^ cm^−1^) using a UV-2550 spectrophotometer (Shimadzu, Japan). The kinetic constants were obtained through nonlinear regression based on the Michaelis–Menten equation (GraphPad Prism 8.0).

### Homology Modeling and Docking Analysis

The homology model of *Ac*RedAm was constructed based on the X-ray structure of *Asp*RedAm (PDB code: 5G6S), using SWISS-MODEL. The docking of the NADPH cofactor, substrates, or product rasagiline into the simulation structure of *Ac*RedAm was performed using the Glide SP program in the Schrödinger package. The residues were evaluated and analyzed using Molecular Operating Environment (MOE) software.

### Library Construction and Expression

Site saturation mutagenesis libraries were constructed by overlap extension PCR with primers containing NNK degenerate codons (Horton et al., 2013), which are listed in Supplementary Section S6. All transformed libraries were plated to single colony density on LB agar plates containing 50 μg/ml kanamycin and grown overnight at 37°C. To obtain the other 19 amino acid exchange mutants from each library, single colonies were picked and confirmed by sequencing. Expression and purification of the confirmed mutants were performed as previously described.

## Results and Discussion

### Computational Exploration of the Novel RedAms Basing on the Sequence and Structure

**FIGURE 1:**
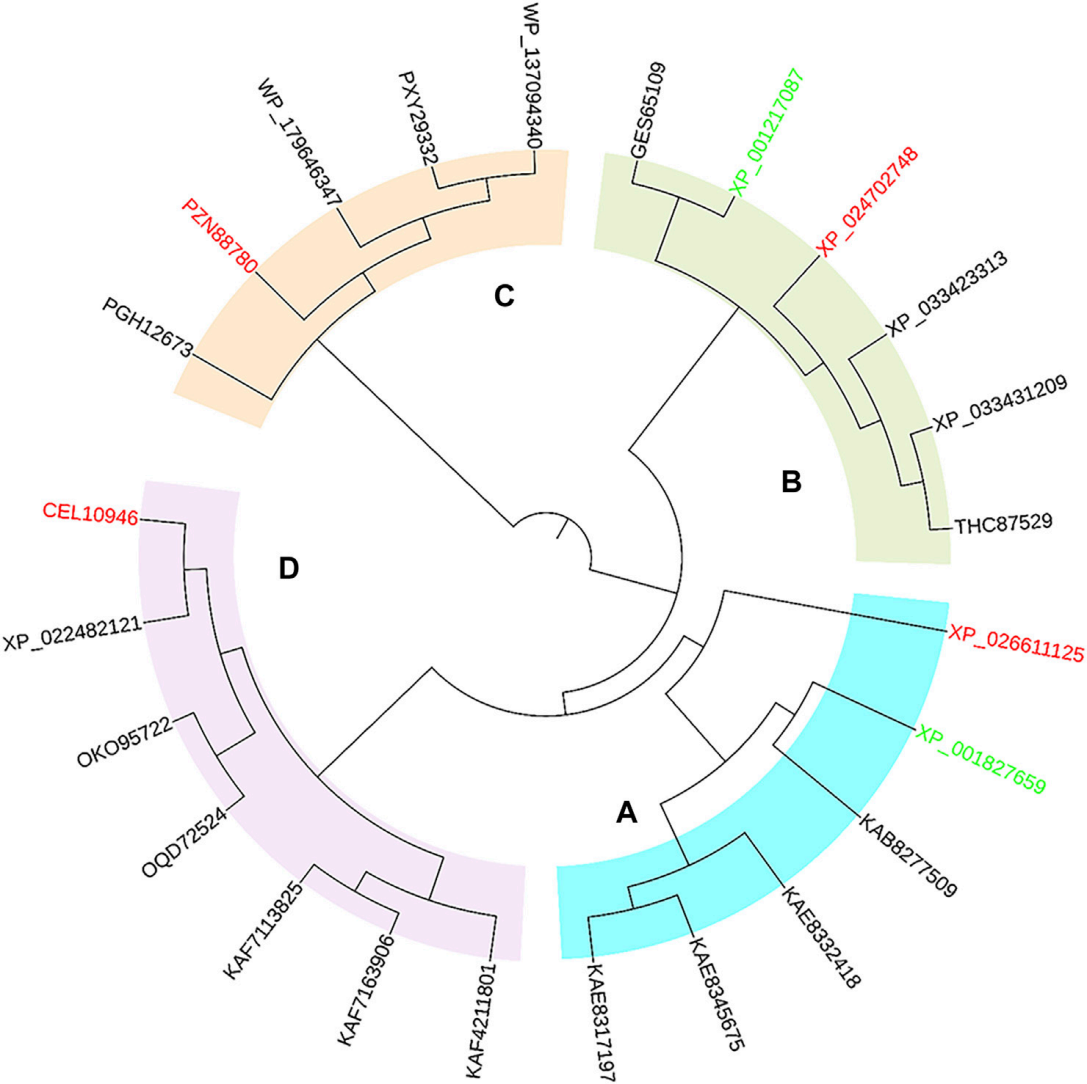
Phylogenetic tree of *Asp*RedAm, *At*RedAm, and potential RedAms. Four major clades were observed, in which four sequences from distinct clades were selected as candidates. The start sequences and candidate sequences are highlighted in green and red, respectively.

Protein exploration based on inquiring sequences and structures is an effective strategy to discover novel enzymes from gene or protein databases. As representative reductive aminases, *Asp*RedAm and *At*RedAm have been well studied for their structure and catalytic mechanism (Aleku et al., 2017; Sharma et al., 2018). To inform the survey, the sequences of *Asp*RedAm (accession no. XP_001827659) and *At*RedAm (accession no. XP_001217087) were used as query sequences for a BLAST search of the databases. Bioinformatics filters (identity >45% and <90%; align length >90%; containing key motifs of RedAms; host of different microorganisms) were used to recruit potential protein sequences. The results of the homologous sequences were then processed by phylogenetic analysis (Figure 1). The phylogenetic tree consisted of four major clades, in which the starting sequences *Asp*RedAm and *At*RedAm belonged to clades A and B, respectively.

Subsequently, a systematic computational analysis of the sequence and structural characteristics of proteins in each major clade was conducted to predict the activity and structure-related enzymatic properties of these proteins. The protein of each major clade was submitted to SWISS-MODEL to model their three-dimensional structures, and then each simulated structure was aligned to the crystal structure of *Asp*RedAm (PDB code: 5G6S). Three reductive aminases from eukaryotic sources and one from bacteria were finally identified based on their similar folding and conservative active pockets to *Asp*RedAm through structural alignment. *As*RedAm (from *Aspergillus steynii*, accession no. XP_024702748), *Ac*RedAm (from *Aspergillus calidoustus*, accession no. CEL10946), *Ath*RedAm (from *Aspergillus thermomutatus*, accession no. XP_026611125) and *Ba*RedAm (from bacterium, accession no. PZN88780) displayed 63, 62, 54, and 46% overall sequence homology with *Asp*RedAm, respectively. As potential RedAm targets, they all possessed conserved residues such as N93, D169, Y177, and Q240 in *Asp*RedAm, which were suggested to be important in catalysis in the template model (Supplementary Figure S1) (Aleku et al., 2017; Sharma et al., 2018). Furthermore, the phylogenetic relationships of four candidates with known IREDs and RedAms revealed that they belonged to the subclass of IREDs, which might possess the capability for reductive amination reactions (Supplementary Figure S2). In the following experiments, the genes of the target RedAms were cloned and expressed in *E. coli* BL21 (DE3); *Ac*RedAm from *Aspergillus calidoustus*, and *Ba*RedAm from bacteria displayed high levels of soluble expression, but the other two were expressed in an insoluble form in inclusion bodies (Supplementary Figure S4). Therefore, soluble *Ac*RedAm and *Ba*RedAm were purified by Ni-NAT affinity chromatography for *in vitro* enzymatic assays.

### Combinatorial Biosynthesis for Chiral Amines Conducted by the New Explored *Ac*RedAm and *Ba*RedAm

**FIGURE 2:**
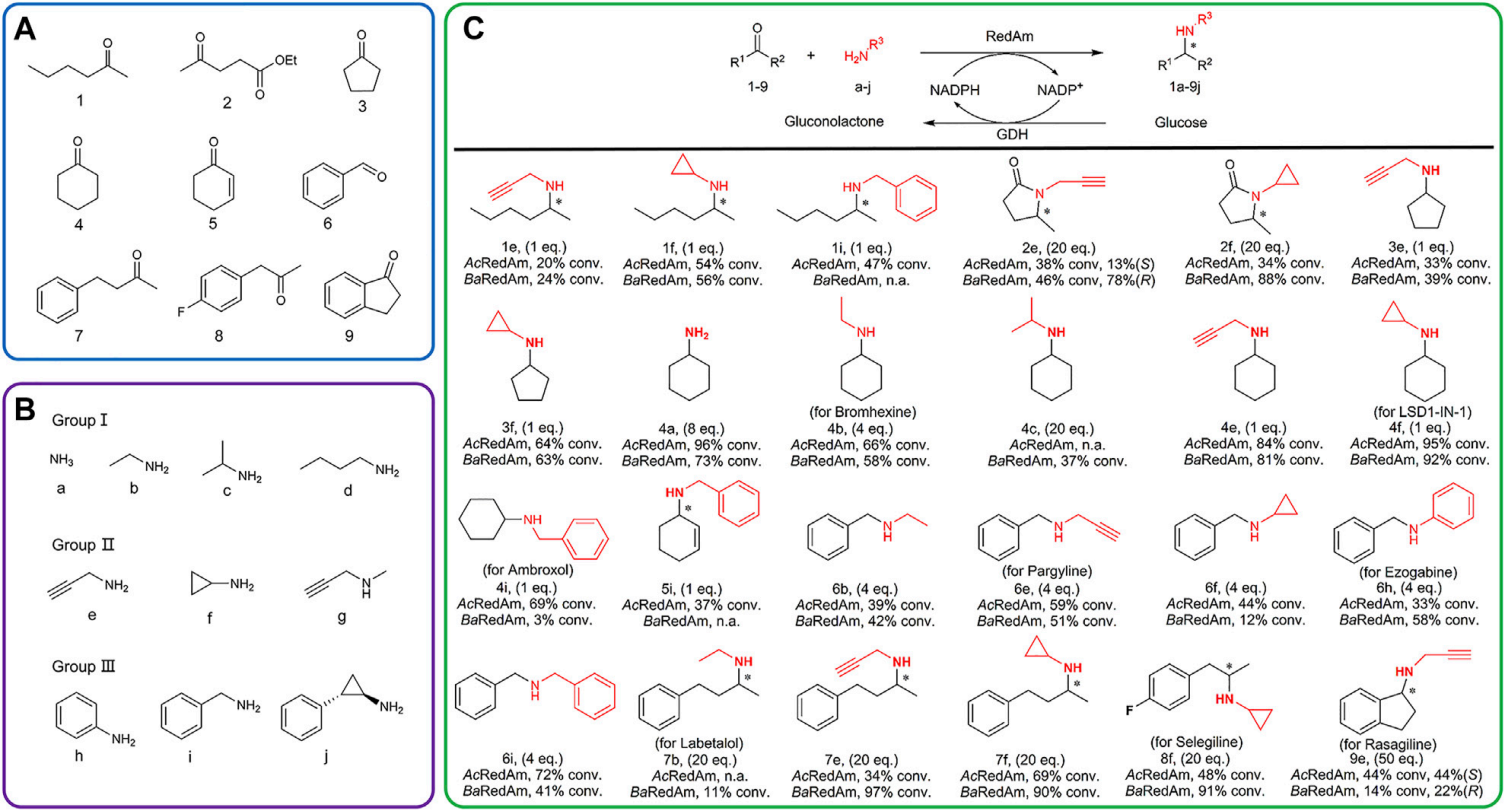
Selected ketones **(A)** and amines **(B)** for the substrate screening of RedAms. **(C)** RedAm-catalyzing reductive amination using selected substrates to access different amines. Reaction conditions: ketone/aldehyde (5 mM), amine (1–50 eq.), RedAm (1 mg ml^−1^), NADP^+^ (1 mM), GDH (0.7 mg ml^−1^), D-glucose (30 mM), and Tris–HCl buffer (100 mM, pH 9.0), 25°C, 220 rpm, 24 h. Conversion determined by HPLC or GC-FID analysis.

A panel of structurally diverse ketones 1-9 and amines a-j was selected to study the reductive amination activity of the novel enzymes (Figures 2A,B). The ketones and amines were arranged according to the properties of their chains. The substrate scope of *Ac*RedAm and *Ba*RedAm was initially assessed by combining a set of ketones (1–9) with different amines. A clear preference for amines e and f was observed (Supplementary Table S2). Subsequently, enzyme-catalyzed reductive amination reactions were conducted with the substrates in an appropriate ratio (Figure 2C). Reductive amination reactions with both aliphatic ketones and aromatic ketones catalyzed by these two RedAms afforded amine products with moderate to excellent conversion (Figure 2C).

In several cases, even equimolar concentrations of ketone and amine resulted in high conversion of 81–95% (Figure 2C, products 4e, 4f); hexan-2-one 1 and cyclopentanone 3 were aminated with one equivalent of e, f, or i by each enzyme with a conversion between 20 and 64%; one equivalent of benzylamine i aminated with ketone 4 or 5 yielded 3–69% conversion to the secondary amine, which was indicative of the genuine reductive amination capacity of these enzymes (Sharma et al., 2018). Ester 2 was aminated with 20 equivalents of e or f by each enzyme with a conversion between 34 and 88%.

In the case of 2e, enantiomeric excess (ee) values between 13 and 78% were observed. Ammonia a was also directly accepted as a donor, giving 96% and 73% conversion by *Ac*RedAm and *Ba*RedAm when coupled with carbonyl acceptor 4, respectively. Benzaldehyde 6 gave secondary amines with 4 equivalents of b, e, f, h, or i, with a conversion of 12–72%. In the presence of some carbonyl acceptors (e.g., 2, 7, and 8), amination with various amines, including ethylamine b, propargylamine e, and cyclopropylamine f, proceeded with higher conversion of up to 97% when using *Ba*RedAm compared to *Ac*RedAm. Products 4a, 4b, 4f, 4i, 6e, 6h, 7b, and 8f could also be used as relevant scaffolds for the manufacture of some pharmaceuticals (Figure 2C). Furthermore, *Ac*RedAm and *Ba*RedAm were both able to directly produce rasagiline 9e starting from 1-indanone 9 and propargylamine e in 44% conversion (44% ee (*S*)-rasagiline) and 14% conversion (22% ee (*R*)-rasagiline), respectively.

### Rational Engineering of RedAms for Enantiospecific Improvement of the Rasagiline Synthesis

The initial results not only suggested that *Ac*RedAm and *Ba*RedAm displayed activity for producing rasagiline 9e starting from 1-indanone 9 and propargylamine e but also prompted in-depth engineering of these enzymes for the synthesis of enantiopure rasagiline as the pharmaceutical ingredient (*R*)-rasagiline is an anti-Parkinson’s agent, and (*S*)-rasagiline provides prominent cardioprotective activity. *Ac*RedAm was first selected for engineering because it afforded higher conversion to rasagiline 9e than *Ba*RedAm.

**FIGURE 3:**
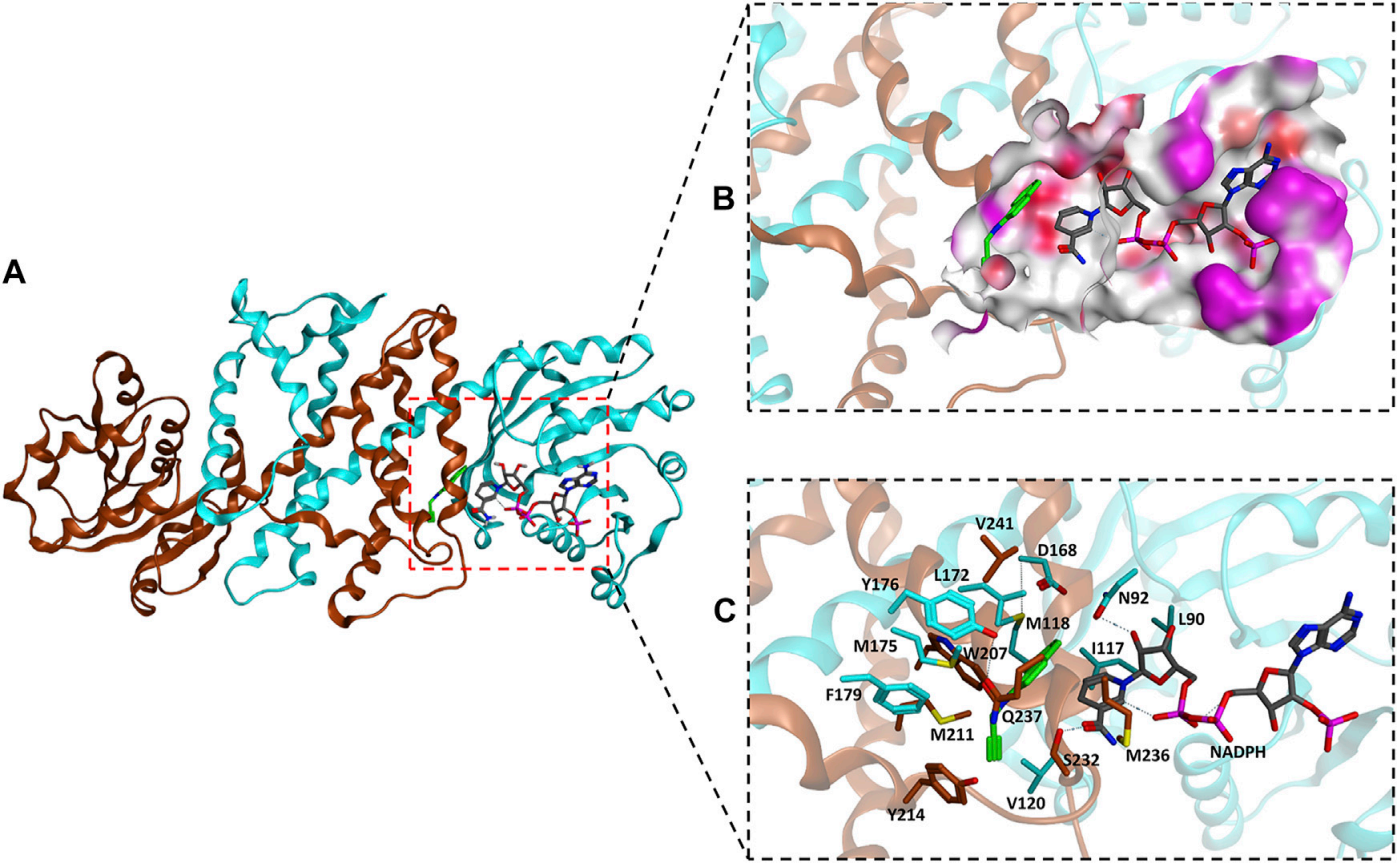
**(A)** Homology model of the *Ac*RedAm dimeric structure in complex with NADPH (gray) and the iminium intermediate of 9e (green) incorporated by docking. **(B)** The binding pocket of *Ac*RedAm is shown, which is at the dimer interface (red, polar; white, hydrophobic; magenta, exposed). **(C)** The residues located within 8 Å of the iminium intermediate are shown as sticks. Carbon atoms of subunits A and B are shown in cyan and orange, respectively.

The homology model of *Ac*RedAm was generated by SWISS-MODEL using the crystal structure of *Asp*RedAm (PDB code: 5G6S) as the template, and the iminium intermediate of 9e and cofactor NADPH were docked to determine residues near or in the binding pocket (Figures 3A,B). The residues located within 8 Å of the iminium intermediate were obtained (Figure 3C). By limiting the analysis of this subset of residues, two amino acids (L90 and I117) and five amino acids (L172, W207, Y214, M236, and Q237) might potentially affect amine e and 1-indanone 9 binding and are involved in product recognition, respectively (Figure 3C; Supplementary Figure S8). Therefore, these 7 amino acids were targeted for single-site saturation mutagenesis. The remaining positions were discounted because they (N92, D168, and Y176) might have important roles in catalysis according to the structure alignment of *Ac*RedAm and *Asp*RedAm. M118 forms a hydrogen bond with the carbon atom of amine e, and others are far from the substrates compared to the target amino acids (Supplementary Figure S8).

### Mutant Characterization of RedAms for the Synthesis of Enantiopure Rasagiline

**FIGURE 4:**
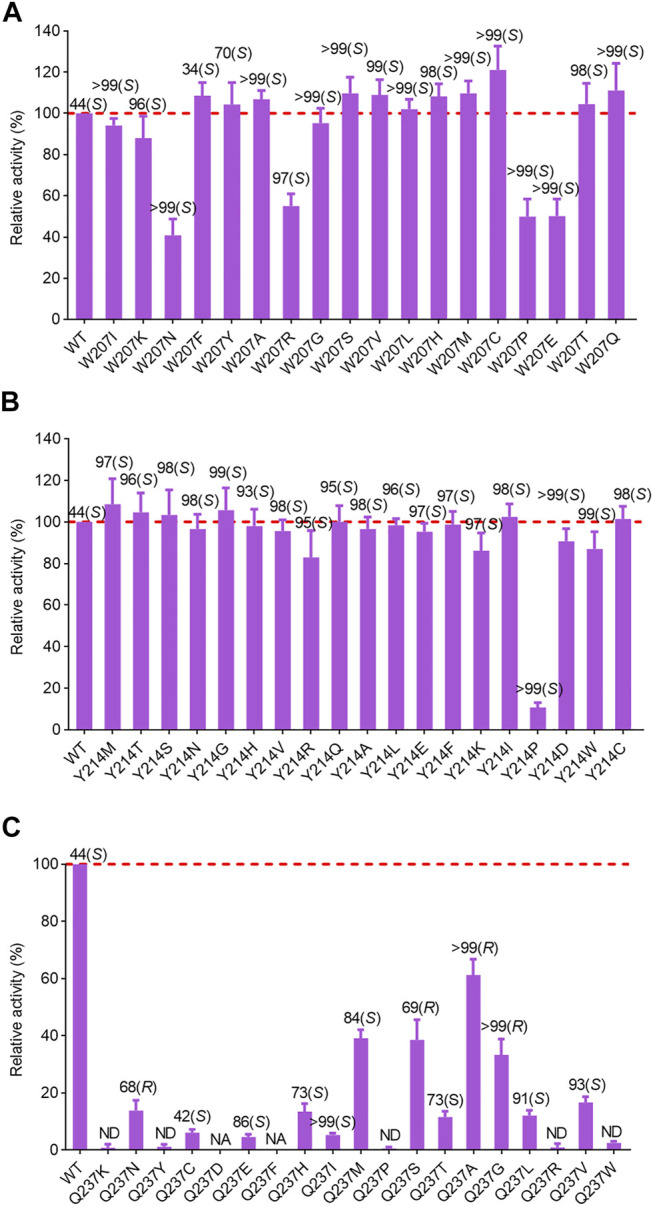
Relative activity and ee values of saturation variants from site 207 **(A)**, 214 **(B)** and 237 **(C)** of *Ac*RedAm for the synthesis of rasagiline. Mutant W207D formed insoluble inclusion bodies. NA indicates no activity; ND indicates not determined for ee value. The activity of the wild-type enzyme was set as 100%. Error bars represent the standard deviations of three replicates.

After identification of the saturation mutagenesis at position 90, a total of 10 variants were purified with soluble expression, indicating that position 90 is a key residue influencing the correct folding of *Ac*RedAm and has a slight effect on the enantioselectivity of the product rasagiline (Supplementary Figure S9). Other positive mutations were identified at positions 207 and 214, with several variants showing slightly improved activity and most of the variants with a high degree of enantioselectivity toward the enantiomer (*S*)-9e (up to >99% ee) (Figures 4A,B). W207C showed the highest activity, which was 1.2-fold that of the wild-type enzyme. In case of single-site saturation mutations at positions 117, 172, 236, and 237, most of the mutants exhibited decreased activity, but several variants such as L172V, Q237N, Q237S, Q237G, and Q237A had moderate to excellent enantioselectivity toward the desired enantiomer (*R*)-9e, with values of 40% ee up to >99% ee (Figure 4C; Supplementary Figure S9).

**FIGURE 5:**
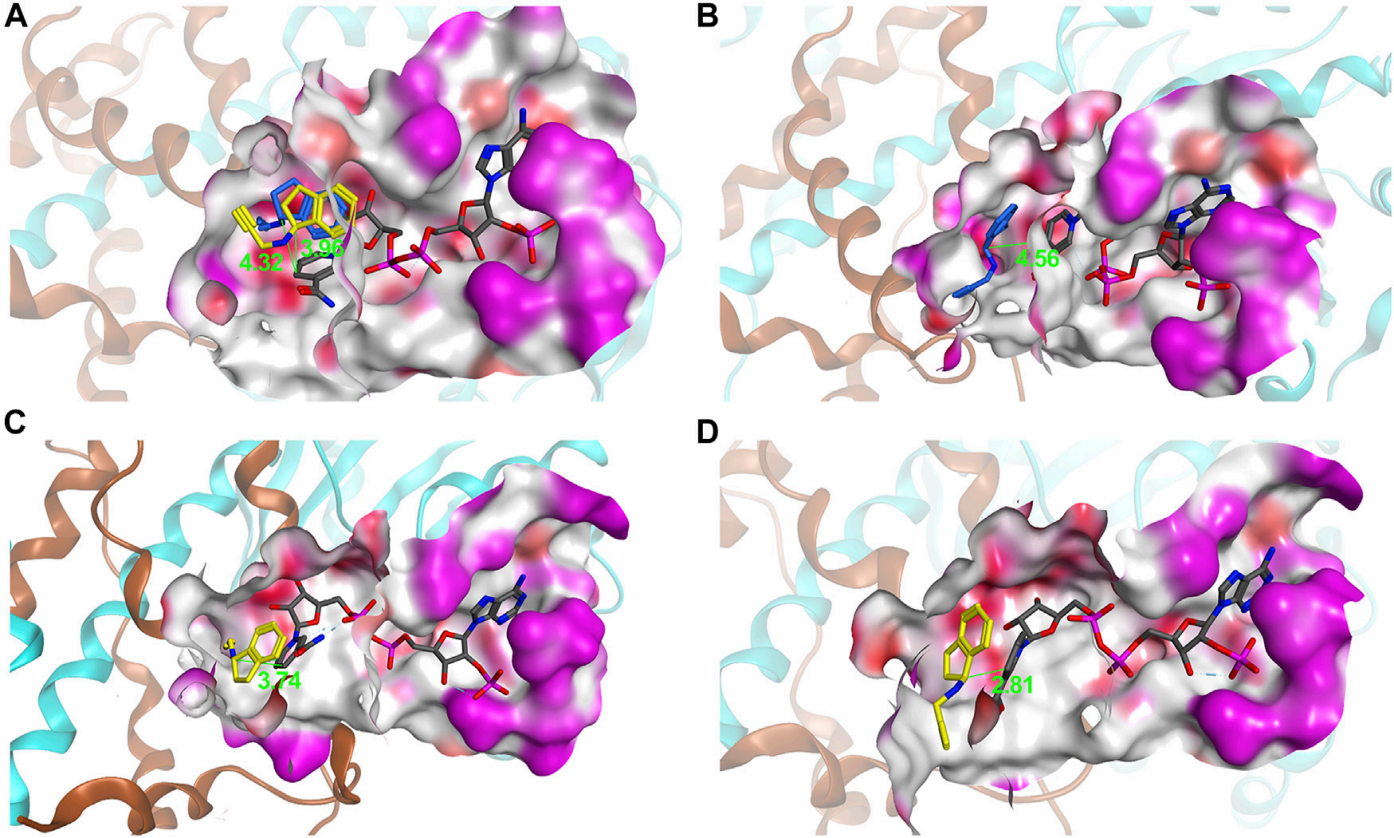
Binding pockets (red, polar; white, hydrophobic; magenta, exposed) and distance between the reactive carbon atom of 9e and the hydride donating carbon (C4) of the nicotinamide of NADPH. **(A)** *Ac*RedAm; **(B)** *Ac*Q237A; **(C)** *Ac*W207C; **(D)** *Ac*W207S/Y214C. NADPH, (*R*)-rasagiline, and (*S*)-rasagiline are shown in gray, blue, and yellow, respectively.

The best-performing mutant was Q237A, which retained 70% activity and had excellent activity toward (*R*)-9e with values > 99% ee (Figure 4C). To investigate the molecular mechanisms of these beneficial mutations, the product (*S*)- or (*R*)-rasagiline and cofactor NADPH were docked into the binding pocket of mutant W207C and Q237A, respectively. The small side chain of C207 was likely to avoid steric hindrance at the bulky indane ring of (*S*)-9e and accommodate the enantiomer (*S*)-9e, leading to the distance between the reactive carbon atom of (*S*)-9e and the hydride donating carbon (C4) of the nicotinamide of NADPH being reduced, which is conducive to the reduction of the iminium ion intermediate, thereby improving the activity (Figures 5A,C) (Sharma et al., 2018; Wang et al., 2021).

However, the small side chain (from alanine, glycine, or serine) at site 237 could also avoid steric hindrance at the bulky indane ring of (*R*)-9e and could accommodate the enantiomer (*R*)-9e; however, the distance between the reactive carbon atom of (*R*)-9e and the hydride donating carbon (C4) of the nicotinamide of NADPH increased, resulting in decreased reaction efficiency (Figures 5A,B) (Sharma et al., 2018; Wang et al., 2021). Moreover, the corresponding residue Q257 from *Ba*RedAm was selected for single-site saturation mutagenesis to investigate its role. Interestingly, the mutants of Q257 could also confer a complete inversion of enantioselectivity toward rasagiline (Supplementary Figure S11), further proving that the residue plays a key role in RedAms.

**FIGURE 6:**
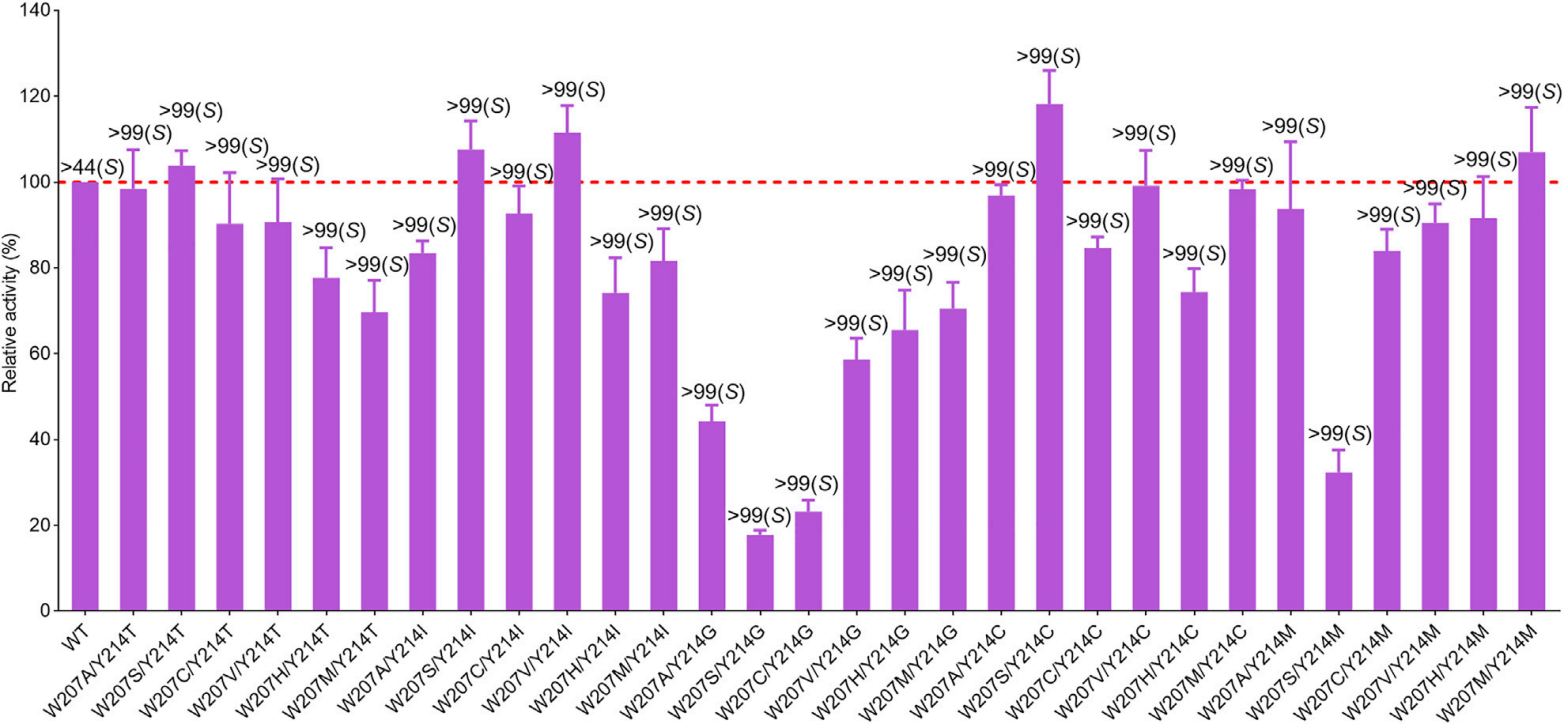
Relative activity and ee values of the double-point mutants for rasagiline synthesis. The activity of wild-type enzyme was set as 100%. Error bars represent the standard deviations of three replicates.

To obtain the optimal mutant with better activity, the mutants showing slightly improved activity were recombined. Although there was no significant increase in activity, all double-point mutants displayed excellent enantioselectivity toward the enantiomer (*S*)-9e (>99% ee) (Figure 6). W207S/Y214C was the best-performing mutant, with a 1.3-fold increase in the activity of the wild-type enzyme. For mutant W207S/Y214C, substitution of these two residues with less polar residues could also avoid steric hindrance at the bulky indane ring of (*S*)-9e and alter the microenvironment of the binding pocket to accommodate the enantiomer (*S*)-9e; the distance between the reactive carbon atom of (*S*)-9e and the hydride donating carbon (C4) of the nicotinamide of NADPH was reduced, thereby improving the activity (Figures 5A,D).

Determination of the kinetic parameters showed that both Q237A and Q237G displayed a decrease in *K*_m_ and *k*_cat_ for indanone 9 compared to the wild type, which indicated that they had increased affinity but decreased activity for substrate 9 (Table 1). In contrast to the results for both Q237A and Q237G mutants, W207C and W207S/Y214C showed a slight increase in *K*_m_ and *k*_cat_ for ketone 9 compared to the wild type, suggesting that they exhibited increased activity but decreased affinity for substrate 9 (Table 1).

**TABLE 1:** Kinetic constants of *Ac*RedAm and its variants toward 1-indanone 9.

Enzyme	*K*_m_ (mM)	*k*_cat_ (s^−1^)	*k*_cat_/*K*_m_ (s^−1^mM^−1^)
Wild type	0.016 ± 0.004	0.017 ± 0.001	1.063 ± 0.041
Q237A	0.009 ± 0.001	0.007 ± 0.001	0.778 ± 0.021
Q237G	0.004 ± 0.001	0.002 ± 0.001	0.500 ± 0.030
W207C	0.018 ± 0.002	0.022 ± 0.001	1.222 ± 0.044
Y214M	0.012 ± 0.004	0.014 ± 0.002	1.167 ± 0.021
W207S/Y214C	0.021 ± 0.004	0.026 ± 0.002	1.238 ± 0.026

Conditions: 0.002-10 mM 1-indanone 9 concentration, 20 mM propargylamine e, RedAm (5-250 ug), NADPH (0.2 mM), 1%(v/v) DMSO, and Tris buffer (100 mM, pH 9.0).

### Synthetic Potential Evaluation of RedAms for Producing Rasagiline in Large Scale

Rasagiline has been reported to be synthesized in high conversion (up to 91%) using IREDs on a preparative scale but failed to obtain enantiopure products (Matzel et al., 2017). Although the known *Asp*RedAm and its variants were reported to have high enantioselectivity for rasagiline (ee value up to 98%), their potential for the preparative scale has not been explored (Aleku et al., 2017). The approach of enantiopure rasagiline bioproduction has become more attractive.

**FIGURE 7:**
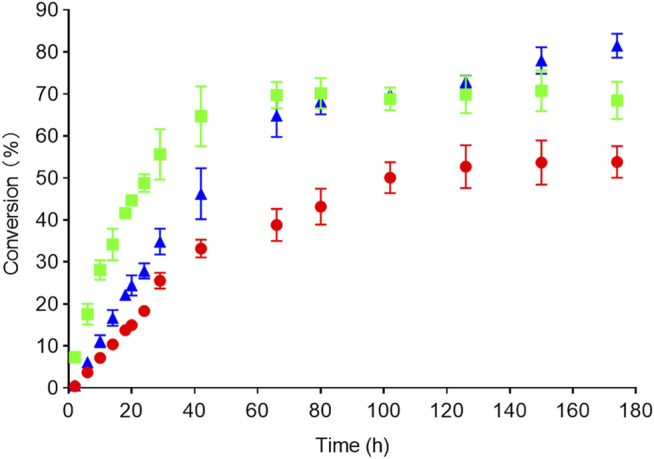
Time course of the reactions. Conversion over time to rasagiline was measured with HPLC and GC (AcRedAm green, Q237A red, BaRedAm blue). Conditions: 5 mM 1-indanone 9, 250 mM propargylamine e, RedAm 1 mg/ml, 100 mM glucose, 1 mM NADP^+^, and 0.7 mg/ml glucose dehydrogenase. Reactions were incubated at 25°C, 220 rpm.

To test the synthetic potential of the candidate RedAms and mutants in this study, a series of large-scale reactions were performed. By applying 9 and e as substrates, the reaction conditions were investigated on an analytical scale prior to conducting the large-scale reaction. The concentrations of ketone, amine, and enzyme loading were investigated as described in detail in Supplementary Section S7 and Supplementary Figure S12. Under optimal conditions, rasagiline 9e was obtained with 70% conversion (yield: 60%) employing *Ac*RedAm after 60 h, and enantiopure (*R*)-rasagiline (ee > 99%) was also successfully synthesized with the Q237A variant on a large scale to afford a product with a conversion of 51% (yield: 42%) after 120 h (Figure 7). Interestingly, 83% conversion (yield: 72%) was achieved using *Ba*RedAm for 180 h, which was consistent with the previous result that *Ba*RedAm possessed greater thermal stability than *Ac*RedAm (Figure 7 and Supplementary Figure S7).

## Conclusion

In summary, the exploration and characterization of two new reductive aminases, *Ac*RedAm, from *A. calidoustus*, and *Ba*RedAm, from bacteria are reported. Both showed a broad substrate scope and could directly produce primary and secondary amines, including some pharmaceutically relevant scaffolds and valuable amine enantiomer rasagiline in a one-pot reaction. Moreover, *Ba*RedAm displayed greater thermostability than the previously reported RedAms, which highlights its potential as a biocatalyst in industrial processes. *Ac*RedAm was successfully engineered for the synthesis of the pharmaceutically enantiopure rasagiline through rational design. Some key residues were identified, which could confer a significant improvement or a complete inversion of enantioselectivity toward rasagiline by a single-site mutant of *Ac*RedAm, such as W207, Y214, and Q237. Finally, the synthetic potential of rasagiline synthesis was explored through a large-scale reaction using *Ac*RedAm, *Ba*RedAm, or *Ac*RedAm Q237A mutants. Taken together, our work paved the way for further engineering other RedAms and developed a biocatalytic toolbox for eco-friendly enzymatic asymmetric synthesis of pharmaceuticals containing chiral amines.

## Data Availability

The datasets presented in the study are included in the article/Supplementary Material, further inquiries can be directed to the corresponding author/s. Additionally, sequence data have been deposited in Genbank with the accession number OL468622 and OL468617.
