# Supplementary material for: Engineering of Reductive Aminases for Asymmetric Synthesis of Enantiopure Rasagiline
Source: Front Bioeng Biotechnol. 2021 Dec 22;9:798147. doi: 10.3389/fbioe.2021.798147 (PMC8727753; doi:10.3389/fbioe.2021.798147)
Supplement: Supplementary file 1 [file DataSheet1.PDF]

Supplementary Material

Engineering of reductive aminases for asymmetric synthesis of enantiopure rasagiline

Kai Zhang, Yuanzhi He, Jiawei Zhu, Qi Zhang, Luyao Tang, Li Cui* and Yan Feng*

State Key Laboratory of Microbial Metabolism, Joint International Research Laboratory of Metabolic and Developmental Sciences, School of Life Sciences and Biotechnology, Shanghai Jiao Tong University, Shanghai, China

Contents

Section 1. General..3
Section 2. Sequence and phylogenetic analysis 3
Section 3. Cloning, expression and purification of the candidate reductive aminases..........................4
Section 3.1 Cloning of RedAms 4
Section 3.2 Expression and Purification of RedAms...5
Section 4. Substrate screening...6
Section 5. Characterization of RedAms 7
Section 6. Directed evolution of RedAms...9
Section 7. Process optimization for large-scale reductive aminations 11
Section 8. Analysis: chromatography columns, conditions and retention times for investigated substrates 12
Section 8.1 HPLC analysis: methods and conditions 12
Section 8.2 GC analysis: methods and conditions..13
Section 9. Chromatograms 14
Section 10. Codon-optimised sequences of candidate RedAms for expression in *E. coli*. 36

Section 1. General

Solvents used were of HPLC grade. Chiral normal phase HPLC was performed on an Agilent system (Santa Clara, CA, USA) equipped with a G1311B binary pump, a G1329B well plate autosampler unit, a G1316A temperature controlled column compartment and a G1314F variable wavelength detector. CHIRALPAK®IC Analytical (Daicel (Osaka, Japan), 250 mm length, 4.6 mm diameter, 5 μm particle size) columns was used. The typical injection volume was 3 μL and chromatograms were monitored at 265 nm. All solvent mixtures are given in (v/v) ratios.

GC analysis was performed on a Agilent 7890B GC (Agilent, Santa Clara, CA, USA) with a flame ionization detector (FID) and autosampler equipped with a 25 m CP-Chirasil-DEX CB column with 0.25 mm inner diameter and 0.25 μm film thickness (Agilent, Santa Clara, CA, USA), HP-5 and HP-INNOWax column (30 m x 0.25 mm x 0.25 μm) from Agilent (Santa Clara, CA, USA).

Section 2. Sequence and phylogenetic analysis

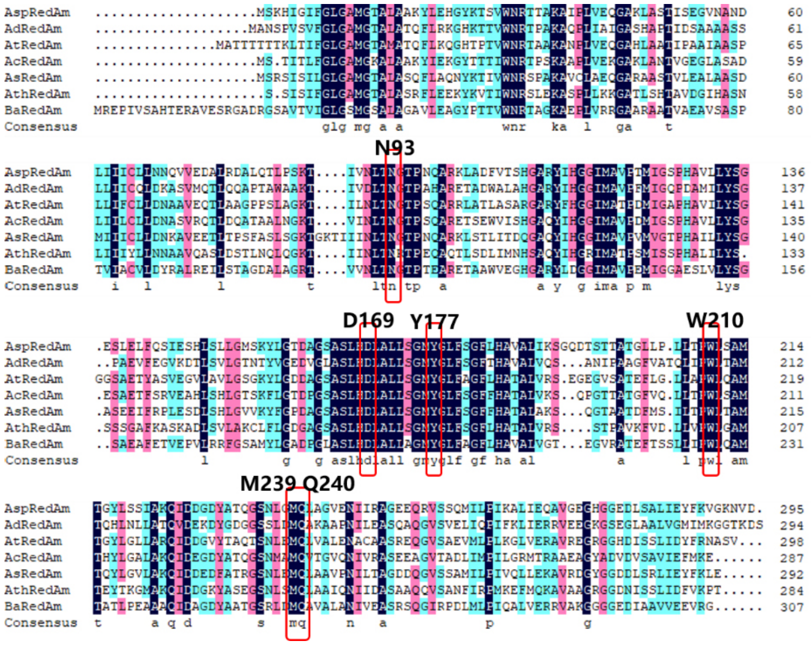

Figure S1. Sequence alignment of the four selected candidates and fungal RedAms. Note: residue (*Asp*RedAm numbering)

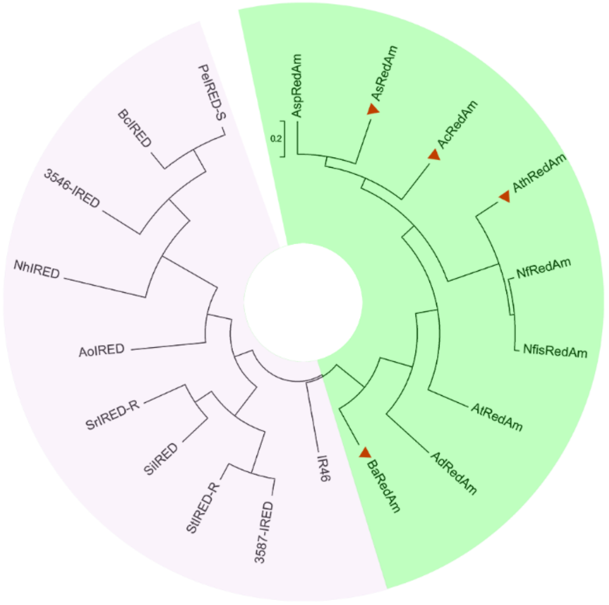

Figure S2. Phylogenetic relationships of 4 candidates in this study with known RedAms and IREDs from the literature.

Section 3. Cloning, expression and purification of the candidate reductive aminases

Section 3.1 Cloning of RedAms

The genes of four candidate RedAms were amplified by PCR using relevant primers (Table S1). The gene fragments were ligated into pET-28a (+) using *Nde*I and *Xho*I restriction sites respectively (Fig. S3). The recombinant plasmids were verified by DNA sequencing, and then transformed into *E. coil* BL21 (DE3) competent cells for gene expression.

Table S1. Primers used for cloning of RedAm genes into pET28a.

Entry	Enzyme	Primers (5' to 3')	Restriction site
1	*Ath*RedAm	GGAATTCCATATGAGCTCAATTTCAAT	*Nde* I
		CCGCTCGAGTTAGGTCGGTTTCACAAAAT	*Xho* I
2	*As*RedAm	GGAATTCCATATGAGCCGTAGCATCAG	*Nde* I
		CCGCTCGAGTTATTCCAGTTTAAAATACTCAA	*Xho* I
3	*Ac*RedAm	GGAATTCCATATGAGCACCATTACCCT	*Nde* I
		CCGCTCGAGTTATTCCTTCATAAACTCG	*Xho* I
4	*Ba*RedAm	GGAATTCCATATGCGTGAACCGATCGT	*Nde* I
		CCGCTCGAGTTAACCGCGCACTTCTT	*Xho* I

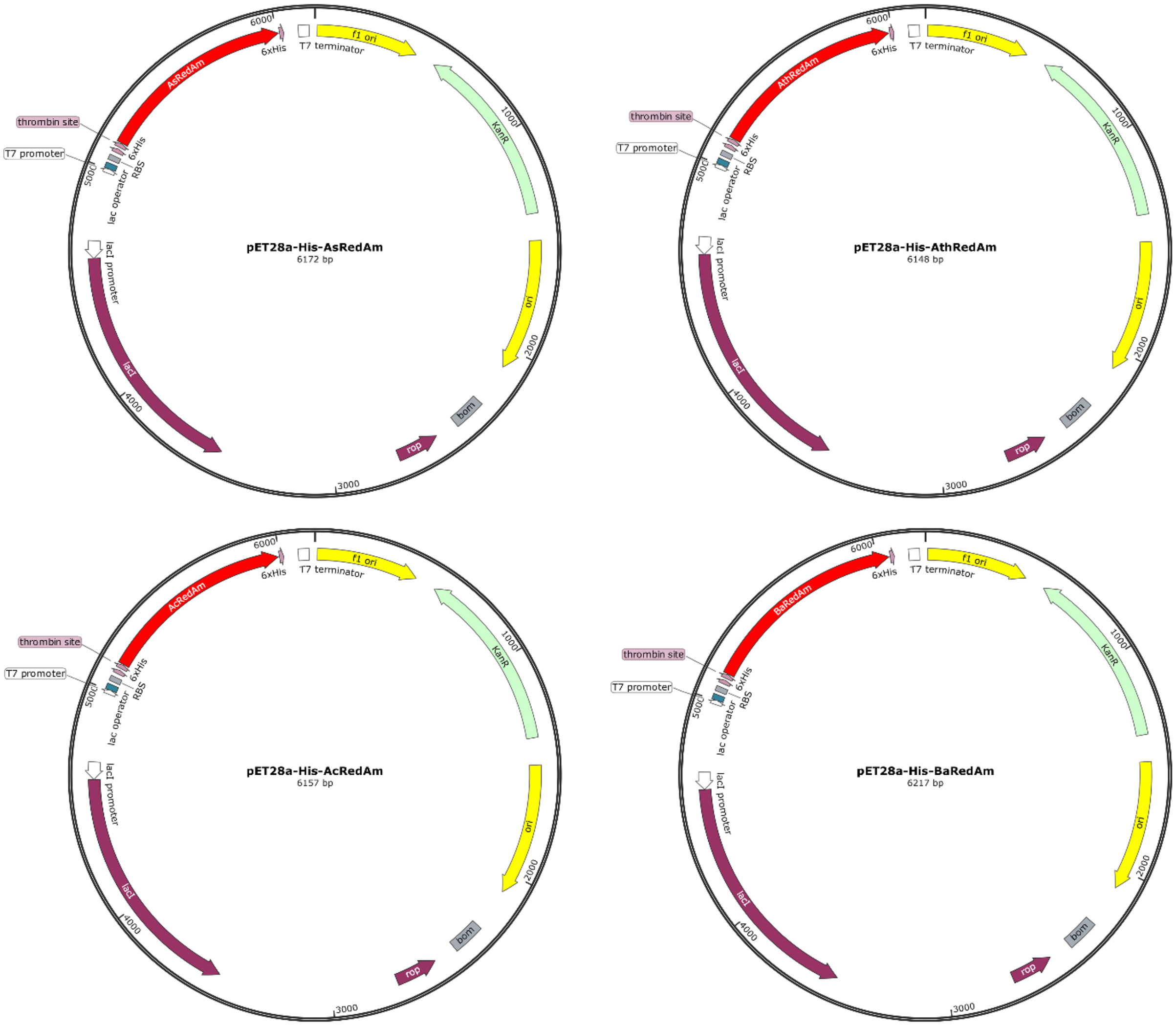

Figure S3. Plasmid map of pET28a-His-*As*RedAm, pET28a-His-*Ath*RedAm, pET28a-His-*Ac*RedAm and pET28a-His-*Ba*RedAm.

Section 3.2 Expression and Purification of RedAms

Single colony was picked from an agar plate grown overnight and cultured in 5 mL LB medium (50 μg/mL kanamycin) at 37°C and 220 rpm for 12 h. Then 2.5 mL preculture was used as the inoculum for shake flask (250 mL LB, 50 μg/mL kanamycin) at 37°C and 220 rpm. At an optical density (OD_{600}) of between 0.6 and 0.8, IPTG was added to a final concentration of 0.5 mM to induce protein expression. Cultivation was continued overnight at 18°C and 220 rpm. The cells were then harvested by centrifugation and resuspended in 100 mM Tris-HCl buffer (pH 8.0, containing 300 mM NaCl and 30 mM imidazole). Following cells disrupted by ultrasonication, the suspension was centrifuged at 10,000 rpm for 30 min to yield a clear lysate. The N-terminal His_6-tagged proteins were purified by Ni-NTA column affinity chromatography. In each case, the lysate was loaded onto a pre-equilibrated Ni-NTA column, followed by washing the non-binding proteins with a load buffer (100M Tris-HCl, 300 mM NaCl, 50mM imidazole pH 8.0). The target protein was eluted with buffer containing 300 mM imidazole. The fractions of target protein were pooled and concentrated using 10 kDa ultrafiltration tube, and the high concentration of imidazole and salt ions were replaced with 100 mM Tris-HCl buffer

(pH 8.0) for 3 times. The protein expression was analyzed by SDS-PAGE. The results are shown in Figure S4. *Ac*RedAm and *Ba*RedAm were overexpressed in completely soluble form and purified, while *Ath*RedAm and *As*RedAm were expressed as inclusion body.

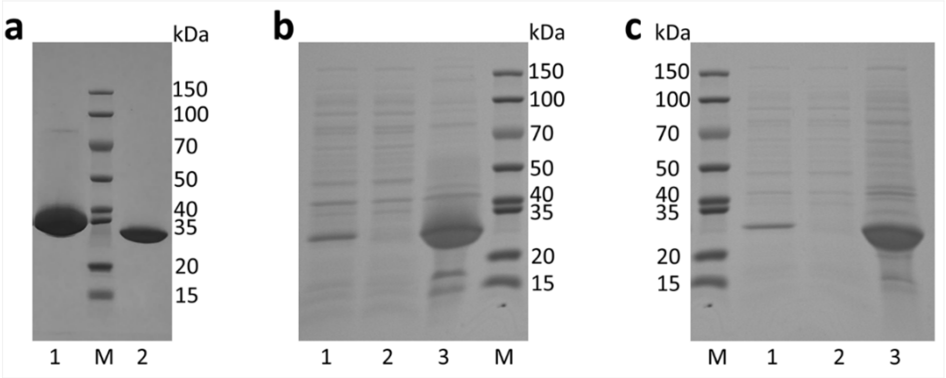

Figure S4. SDS-PAGE analysis of candidate RedAms. (**a**) Lane 1 is the purified *Ba*RedAm; Lane 2 is the purified *Ac*RedAm. (**b**) Expression of *As*RedAm. Lane 1 is the cell lysates; Lane 2 is the supernatant of cell lysates; Lane 3 is the precipitation of cell lysates. (**c**) Expression of *Ath*RedAm. Lane 1 is the cell lysates; Lane 2 is the supernatant of cell lysates; Lane 3 is the precipitation of cell lysates. The loading amount of purified *Ba*RedAm and *Ac*RedAm were 20 ug and 10 ug respectively. For *As*RedAm and *Ath*RedAm, the loading amount of cell lysates was equal.

Section 4. Substrate screening

The screenings were performed in 100 mM Tris-HCl buffer (pH 9.0) containing 1 mg/mL purified RedAm, 1 mg/mL GDH (Aladdin), 30 mM D-glucose, 1 mM $NADP^+$, 5 mM ketone, 100 mM amine (in buffer adjusted to pH 9.0) and 2% (v/v) DMSO. The final reaction volume was made up to 500 ul with Tris-HCl buffer. Reactions were incubated at 30 ℃ with 220 rpm shaking for 24 h. Then, 30 ul of 10 M NaOH was added to quench the reactions. The reaction mixture was extracted twice with 500 ul *tert*-butyl methyl ether. The organic fractions were combined and dried over anhydrous $MgSO_4$ and analyzed by HPLC or GC-FID.

Table S2. Investigated ketones **1-9**, amines **a-j** and resulted products.

	NH_3 **a**	**b**	**c**	**d**	**e**	**f**	**g**	**h**	**i**	**j**
**1**					+	+			+	
**2**					+	+				
**3**					+	+				
**4**	+	+	+		+	+			+	
**5**									+	
**6**		+			+	+		+	+	
**7**		+			+	+				
**8**						+				
**9**					+					

Note: + indicated that amine products formed, others showed no products detected.

Section 5. Characterization of RedAms

Based on the initial screening result, the reductive amination of cyclohexanone **4** and cyclopropylamine **f** at equimolar concentrations was selected as a model reaction to characterize RedAms as both of them show high activity for this transformation. The optimal pH for reductive amination of these two RedAms were measured at different pH in 100 mM Tris-HCl buffer. The optimal temperature for reductive amination of these two RedAms were measured at different temperatures (Fig. S5). Optimal reductive amination activity was observed at pH 9.0 and 25 °C. The concentrations of DMSO between 2% and 10% did not have a dramatic effect on the activities of these two RedAms (Fig. S6).

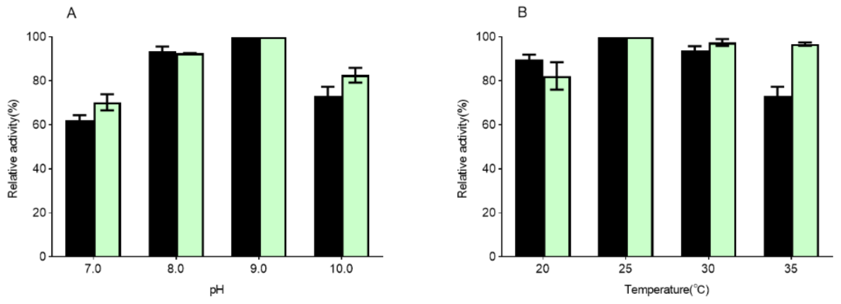

Figure S5. Effects of pH and temperature on the activities of *Ac*RedAm (black) and *Ba*RedAm (green). Relative activity was expressed as a percentage of the maximum activity.

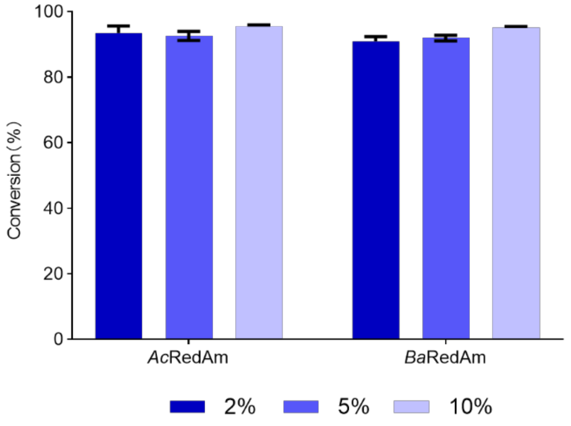

Figure S6. Effects of different DMSO concentrations on the activities of *Ac*RedAm and *Ba*RedAm.

Kinetic assays were performed using the method as previously reported in Aleku et al. For the determination of kinetic parameters of these two RedAms for cyclohexanone **4**, a typical reaction mixture contained 0.2-60 mM of ketone **4**, 100 mM cyclopropylamine **f** from buffer stock adjusted to pH 9.0, 0.4 mM NADPH, 1% DMSO and purified enzyme with appropriate concentration in a total volume of 200 ul (100 mM Tris-HCl, pH 9.0). Activity measurements were performed in triplicate at 340 nM (ε = 6.22 mM^{-1} cm^{-1}) by using UV-2550 spectrophotometer (Shimadzu, Japan). The kinetic constants were obtained through nonlinear regression based on Michaelis-Menten equation (GraphPad Prism 8.0).

Table S3. Kinetic parameters for cyclohexanone **4** in the reductive amination of cyclohexanone **4** and cyclopropylamine **f** .

RedAm	K_M(mM)	k_{cat}(s^{-1})	k_{cat}/K_M ($s^{-1}mM^{-1}$)
*Ac*RedAm	1.59±0.21	1.33±0.03	0.84
*Ba*RedAm	5.55±1.45	5.52±0.28	0.99

Conditions: 0.2-60 mM cyclohexanone **4** concentration, 100mM cyclopropylamine **f**, RedAm (5-100 ug), NADPH (0.4 mM), 1%(v/v) DMSO, Tris buffer (100 mM, pH 9.0)

In order to further investigate the biophysical characteristics of these two RedAms, the melting temperature (*Tm*) was determined by using differential scanning calorimetry (DSC). The unfolding of the enzymes was studied by recording the heat capacity (Cp) at different temperatures (30-80 °C). The melting temperature of *Ba*RedAm was 67 °C, which showed 22 °C higher than *Ac*RedAm (Fig. S7A). The thermostability of these two enzymes were also evaluated by measuring the residual activity after incubation at 50 °C at different times (Fig. S7B). In this case, the reductive amination of cyclohexanone 4 and cyclopropylamine **f** at equimolar concentrations and 25 °C was selected as a control reaction.

After an incubation time of 60 min, *Ac*RedAm lost most of the activity, whilst *Ba*RedAm still gave 90% conversion to **4f**.

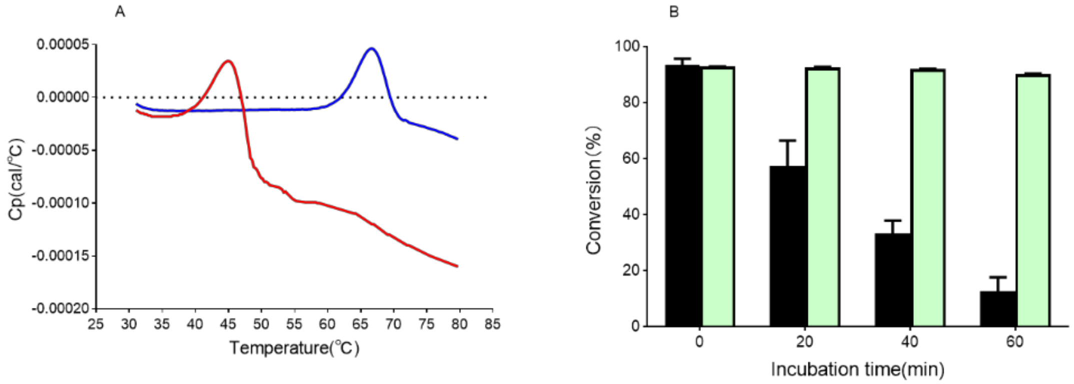

Figure S7. Thermostability studies of RedAms. (**A**) Thermal denaturation studies as a measure of thermodynamic stability. *Ac*RedAm (red), *Ba*RedAm (blue); (**B**) Conversions of *Ac*RedAm (black) and *Ba*RedAm (green) in the equimolar bio-reductive amination of cyclohexanone **4** and cyclopropylamine **f** after incubation at 50 °C at different times.

Section 6. Directed evolution of RedAms

In this section, the recombinant plasmid was used as the template. Site-saturation mutagenesis libraries were constructed by overlap extension PCR with primers containing NNK degenerate codons, which were listed in **Table S4**.

Table S4. Primers used for constructing Site-saturation mutagenesis libraries.

Enzyme	Site	Primers (5’ to 3’)
*Ac*RedAm	L90	AAGACCGTTATCAACNNKACCAACGGTA
		MNNGTTGATAACGGTCTTGCCGTTCA
	I117	TACATCCACGGTGGCNNKATGGCGGTGC
		MNNGCCACCGTGGATGTATTGCGCG
	L172	CACGATCTGGCGCTGNNKAGCGGCATGT
		MNNCAGCGCCAGATCGTGCAGGCTCG
	W207	CAACTGCTGACCCCGNNKCTGAGCGCGA
		MNNCGGGGTCAGCAGTTGAACAAAACCG
	Y214	AGCGCGATGACCCACNNKCTGGGTGCGC
		MNNGTGGGTCATCGCGCTCAGCCAC
	M236	GGCAGCAACATGGCGNNKCAGGTGACCG
		MNNCGCCATGTTGCTGCCTTGGGTC
	Q237	AGCAACATGGCGATGNNKGTGACCGGTG
		MNNCATCGCCATGTTGCTGCCTTGG
*Ba*RedAm	Q257	AGTCGTCTGGATATGNNKGCCGTTGCGC
		MNNCATATCCAGACGACTACCGGTGG

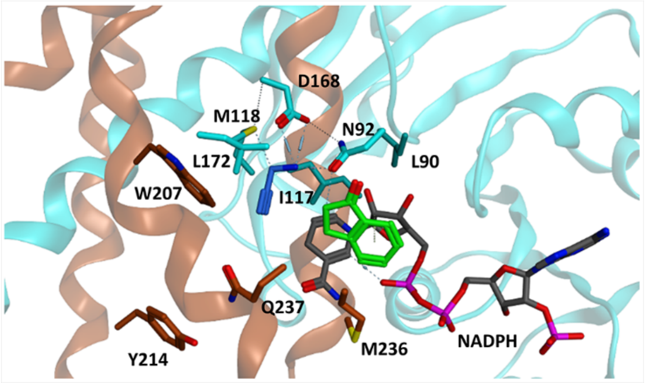

Figure S8. Docking of indanone **9** (green), proparygylamine **e** (blue) and NADPH (gray) into *Ac*RedAm, respectively. Redsidues are shown as sticks.

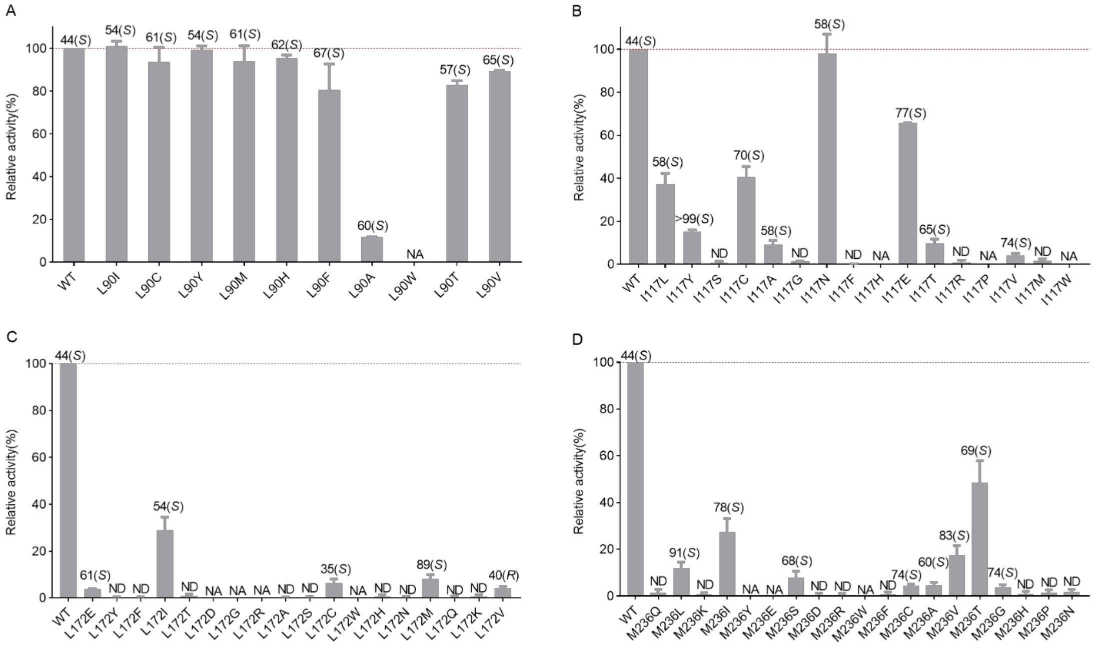

Figure S9. Relative activity and ee value of saturation variants from each selected site of *Ac*RedAm for synthesizing rasagiline. The data of mutants that formed inclusion bodies not shown; NA indicates no activity； ND indicates not determined for ee value. The activity of wild-type enzyme was set as 100%. Error bars represent the standard deviations of three replicates.

For further investigating the role of some residues, the variants exhibiting different enantioselectivity were recombined. However, most of the double-point mutants lost activity, though some variants displayed enantioselectivity towards the enantiomer (*R*)-**9e**, but with poor activities. Among of these double-point mutants, the better active mutants still had enantioselectivity towards the enantiomer (*S*)-**9e** (Fig. S10).

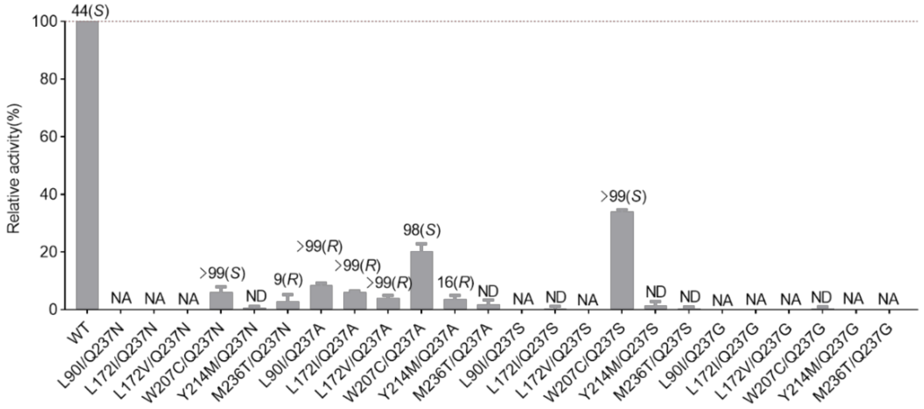

Figure S10. Relative activity and ee value of the double-point mutants for synthesizing rasagiline. NA indicates no activity； ND indicates not determined for ee value. The activity of wild-type enzyme was set as 100%. Error bars represent the standard deviations of three replicates.

For further investigating the role of residue Q237, the homology model of *Ba*RedAm was also constructed based on the X-ray structure of *Asp*RedAm, using SWISS-MODEL. Through sequence-structure alignment with *Ac*RedAm and *Ba*RedAm, the corresponding residue Q257 from *Ba*RedAm was selected for site-directed saturating. The results showed that the residue Q257 could also confer a complete inversion of enantioselectivity towards rasagiline (Fig. S11).

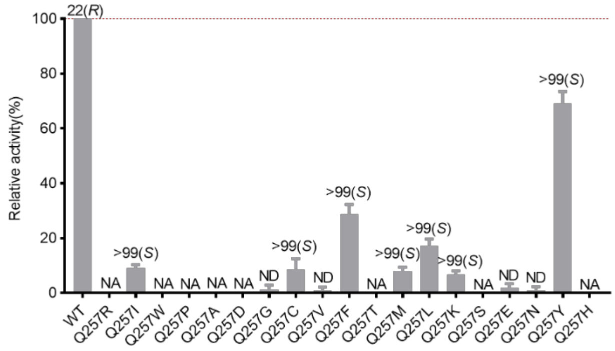

Figure S11. Relative activity and ee value of saturation variants from *Ba*RedAm Q257 site for synthesizing rasagiline. NA indicates no activity；ND indicates not determined for ee value. The activity of wild-type enzyme was set as 100%. Error bars represent the standard deviations of three replicates.

Section 7. Process optimization for large-scale reductive aminations

To optimize the large-scale reactions, different reaction conditions were investigated to obtain high conversion for the product rasagiline **9e**. In analytical scale experiments ketone concentrations were varied from 5 mM up to 50 mM, amine concentrations from 50 mM up to 1 M, GDH (glucose dehydrogenase) concentrations between 0.7 mg/ml and 2 mg/ml and RedAm concentrations between 0.25 mg/ml and 2.5 mg/ml. In order to compare the results, reactions were run using the standard reductive amination conditions, in which only one parameter was varied. The standard conditions were set as follows: 5 mM ketone, 200 mM amine, RedAm 1 mg/ml, 30 mM D-glucose, 1 mM $NADP^+$, 0.7

mg/ml GDH, 2% dimethylsulfoxide, 100 mM Tris buffer pH 9.0, 250 rpm, 24 h. The results indicated that high conversion could be achieved at a low ketone loading with 50 amine equivalents and a moderate enzyme loading. Therefore, these conditions were employed for the large-scale reactions.

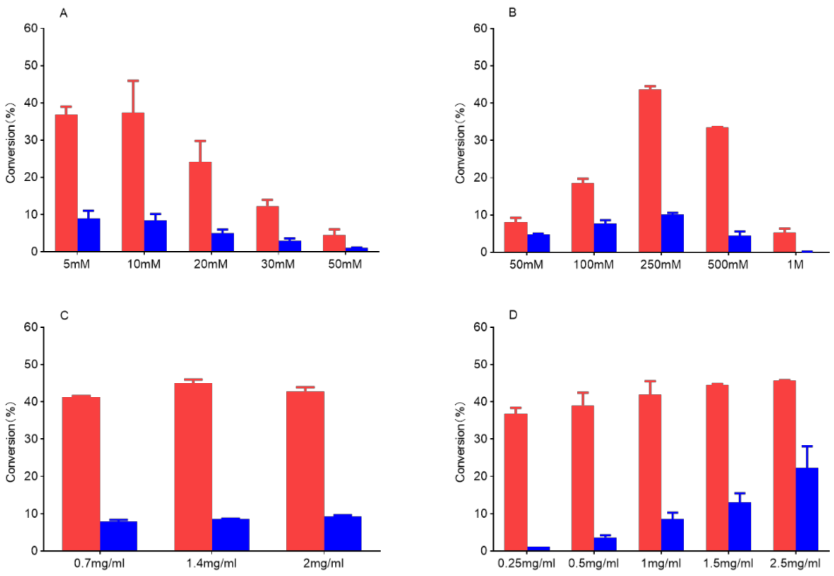

Figure S12: Reaction optimization. (**A**) Optimization of the substrate ketone concentrations; (**B**) Optimization of the substrate amine concentrations; (**C**) Optimization of GDH concentrations; (**D**) Optimization of RedAms concentrations. Conversions after 24 h were measured by HPLC and GC.

Section 8. Analysis: chromatography columns, conditions and retention times for investigated substrates

Section 8.1 HPLC analysis: methods and conditions

The biotransformation products were analyzed by normal phase chiral HPLC using isocratic methods with mobile phase *n*-hexane and isopropanol in different solvent ratios, and 0.1% diethylamine as additive. The flow rate was maintained at 1 mL min^{-1} and elutes were detected by the UV detector at a wavelength of 265 nm.

Table S5. HPLC analysis: methods and retention times for substrates and amine products.

Ketone	Amine donor	Amine product	Column	*n*-hexane/IPA/ diethylamine running solvent ratio	Ketone retention time (min)	Amine product retention time (min)	
6	b	6b	CHIRALPAK®IC	90:10:0.1	8.1	6.1	
6	e	6e	CHIRALPAK®IC	98:2:0.1	8.7	7.2	
9	e	9e	CHIRALPAK®IC	98:2:0.1	27.4	7.2 (*S*)	7.9 (*R*)

Section 8.2 GC analysis: methods and conditions

Table S6. GC analysismethods and retention times of substrates and amine products from biotransformations. Injector temperature: 200 °C, detector temperature: 250 °C, nitrogen flow: 1ml min^{-1}.

Ketone	Amine donor	Amine product	Column	Oven temp.	Ketone retention time/ min	Amine product retention time/ min	
1	e	1e[a]	CP-ChiralSil-DEX CB	50-200°C, 5°C min^{-1}	7.1	13.2	13.3
1	f	1f[a]	CP-ChiralSil-DEX CB	50-200°C, 5°C min^{-1}	7.1	12.2	12.3
1	i	1i	CP-ChiralSil-DEX CB	50-200°C, 5°C min^{-1}	7.1	22.9[b]	
2	e	2e	CP-ChiralSil-DEX CB	100-140°C, 2.5°C min^{-1}, 140-200°C, 10°C min^{-1}	7.3	15.1 (*R*)	15.4 (*S*)
2	f	2f[a]	CP-ChiralSil-DEX CB	100-140°C, 2.5°C min^{-1}, 140-200°C, 10°C min^{-1}	7.3	17.0	17.2
3	e	3e	HP-INNOWax	50-200°C, 5°C min^{-1}	7.0	13.1	
3	f	3f	CP-ChiralSil-DEX CB	50-200°C, 5°C min^{-1}	8.2	13.7[c]	
4	a	4a	HP-5	50-200°C, 5°C min^{-1}	6.5	5.8	
4	b	4b	CP-ChiralSil-DEX CB	50-200°C, 5°C min^{-1}	12.1	13.2[c]	
4	c	4c	HP-5	50-200°C, 5°C min^{-1}	6.4	9.5	
4	e	4e	HP-INNOWax	50-200°C, 5°C min^{-1}	9.3	15.3	
4	f	4f	CP-ChiralSil-DEX CB	50-200°C, 5°C min^{-1}	12.0	16.2[c]	
4	i	4i	CP-ChiralSil-DEX CB	50-200°C, 5°C min^{-1}	12.0	26.5[c]	
5	i	5i[a]	CP-ChiralSil-DEX CB	50-200°C, 5°C min^{-1}	13.1	26.5	27.2
6	f	6f	CP-ChiralSil-DEX CB	50-200°C, 5°C min^{-1}	12.9	19.5[c]	
6	h	6h	CP-ChiralSil-DEX CB	50-200°C, 5°C min^{-1}	12.8	30.4[c]	
6	i	6i	CP-ChiralSil-DEX CB	50-200°C, 5°C min^{-1}	12.8	29.7[c]	
7	b	7b[a]	CP-ChiralSil-DEX CB	50-154°C, 2°C min^{-1}, 154-200°C, 10°C min^{-1}	39.3	42.8	42.9
7	e	7e	CP-ChiralSil-DEX CB	50-154°C, 2°C min^{-1}, 154-200°C, 10°C min^{-1}	39.3	51.2[b]	
7	f	7f	CP-ChiralSil-DEX CB	50-154°C, 2°C min^{-1}, 154-200°C, 10°C min^{-1}	39.3	49.5[b]	
8	f	8f	CP-ChiralSil-DEX CB	50-154°C, 2°C min^{-1}, 154-200°C, 10°C min^{-1}	35.8	44.3[b]	
9	e	9e	HP-INNOWax	50-200°C, 5°C min^{-1}	25.4	28.8	

[a] absolute configuration not determined. [b] separation of entiomers was not achieved. [c] Non-chiral product.

Section 9. Chromatograms

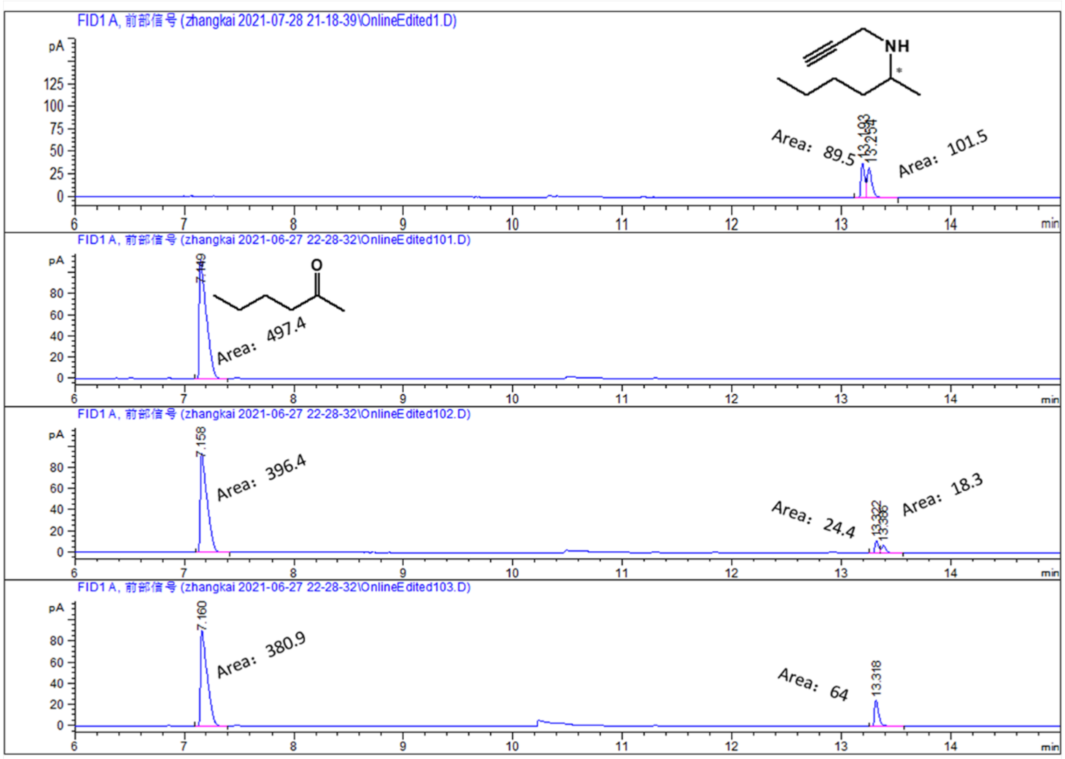

Figure S13. GC analysis: RedAm-catalyzing reductive amination of 2-hexanone **1** with propargylamine **e** (1:1 molar ratio) showing standards of amine product **1e** (top), 2-hexanone **1** (second), biotransformation trace with *Ac*RedAm (third) and biotransformation trace with *Ba*RedAm (bottom).

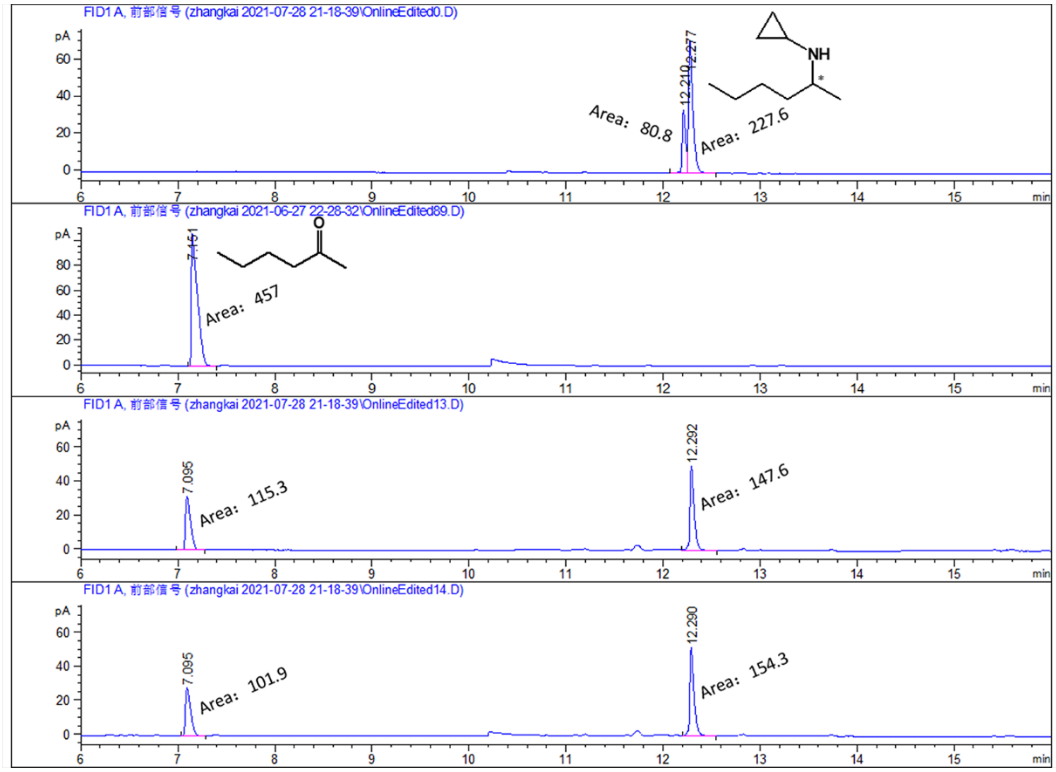

Figure S14. GC analysis: RedAm-catalyzing reductive amination of 2-hexanone **1** with cyclopropylamine **f** (1:1 molar ratio) showing standards of amine product **1f** (top), 2-hexanone **1** (second), biotransformation trace with *Ac*RedAm (third) and biotransformation trace with *Ba*RedAm (bottom).

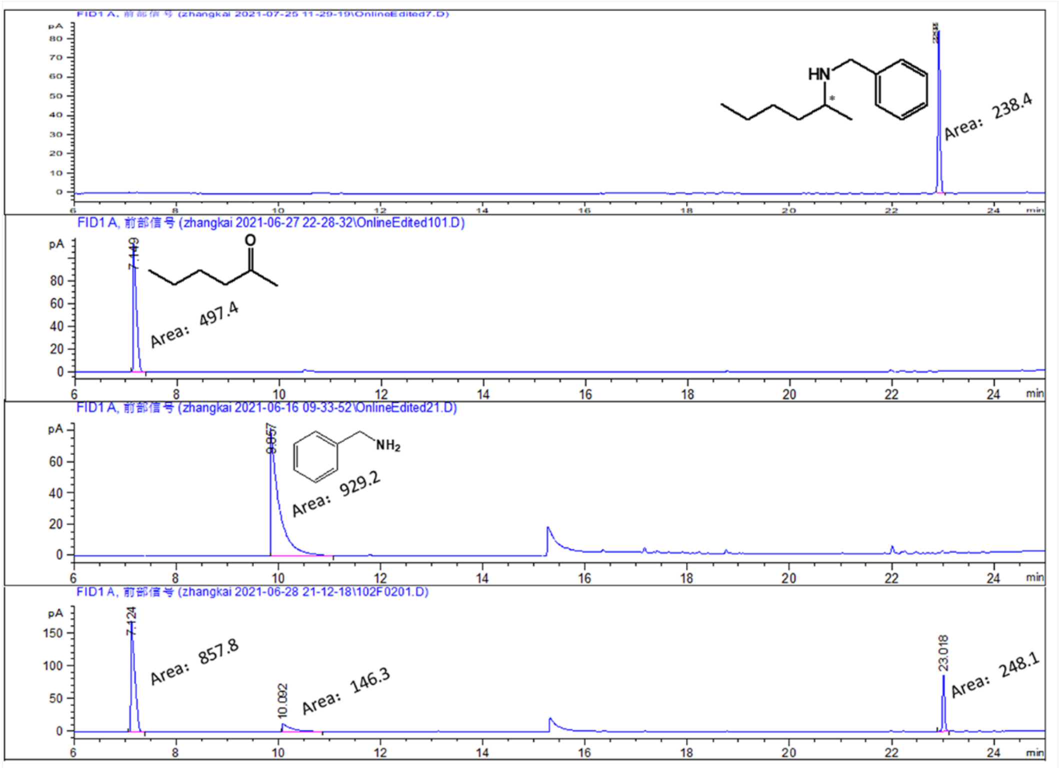

Figure S15. GC analysis: RedAm-catalyzingreductive amination of 2-hexanone **1** with benzylamine **i** (1:1 molar ratio) showing standards of amine product **1i** (top), 2-hexanone **1** (second), benzylamine **i** (third), and biotransformation trace with *Ac*RedAm (bottom).

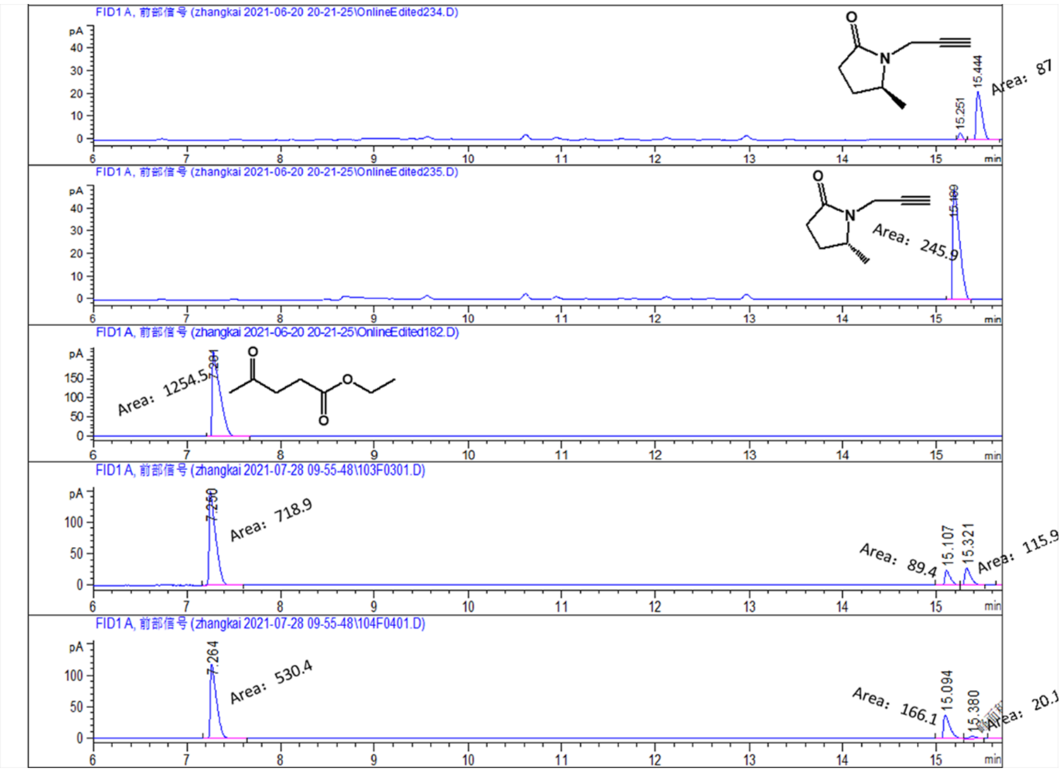

Figure S16. GC analysis: RedAm-catalyzing reductive amination of ketone **2** with propargylamine **e** (1:20 molar ratio) showing standards of **(*S*)-2e** (top), **(*R*)-2e** (second), ketone **2** (third), biotransformation trace with *Ac*RedAm (fourth) and biotransformation trace with *Ba*RedAm (bottom).

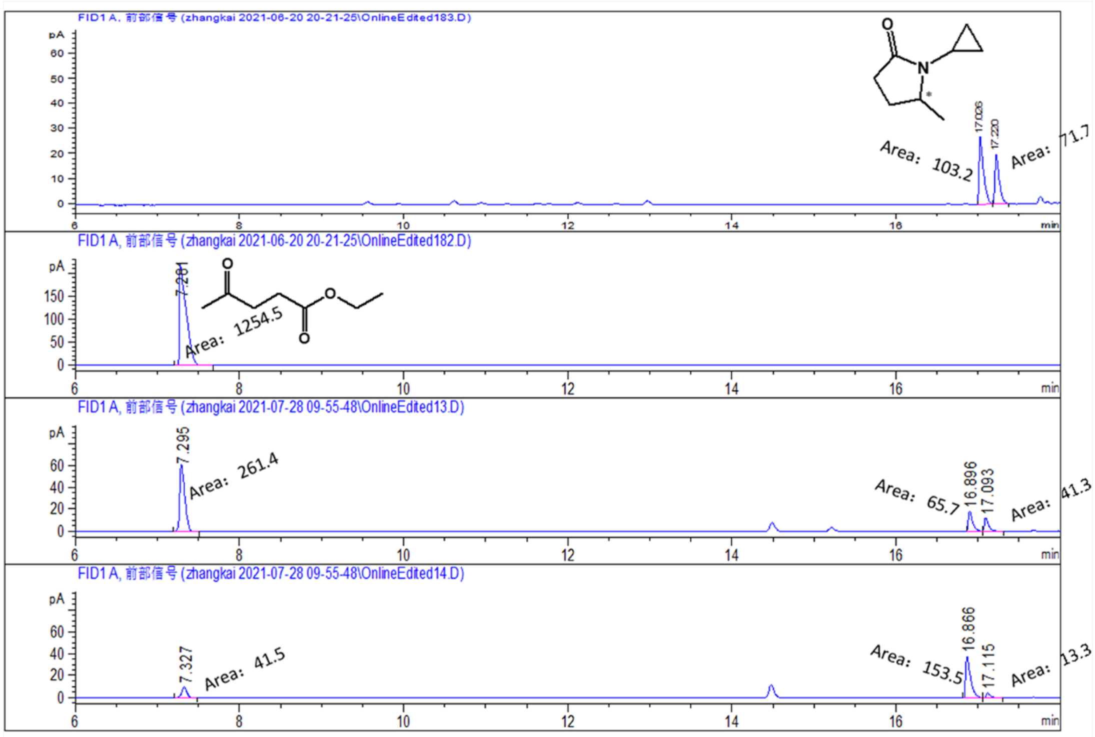

Figure S17. GC analysis: RedAm-catalyzing reductive amination of ketone **2** with cyclopropylamine **f** (1:20 molar ratio) showing standards of amine product **2f** (top), ketone **2** (second), biotransformation trace with *Ac*RedAm (third) and biotransformation trace with *Ba*RedAm (bottom).

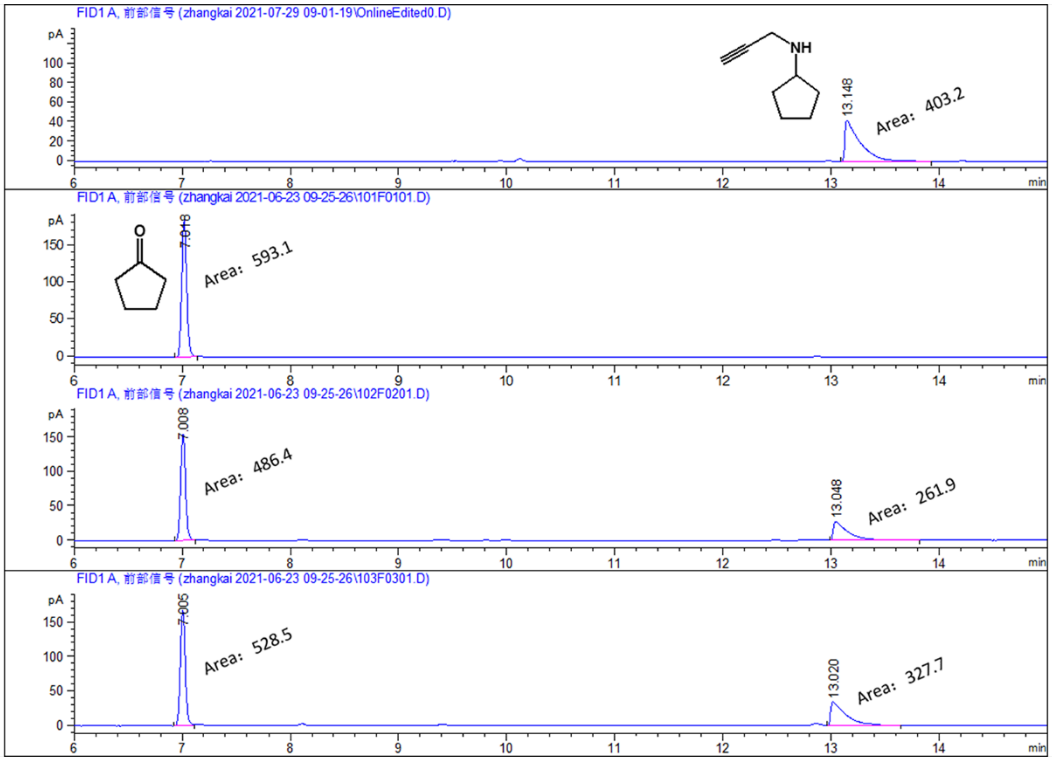

Figure S18. GC analysis: RedAm-catalyzing reductive amination of cyclopentanone **3** with propargylamine **e** (1:1 molar ratio) showing standards of amine product **3e** (top), ketone **3** (second), biotransformation trace with *Ac*RedAm (third) and biotransformation trace with *Ba*RedAm (bottom).

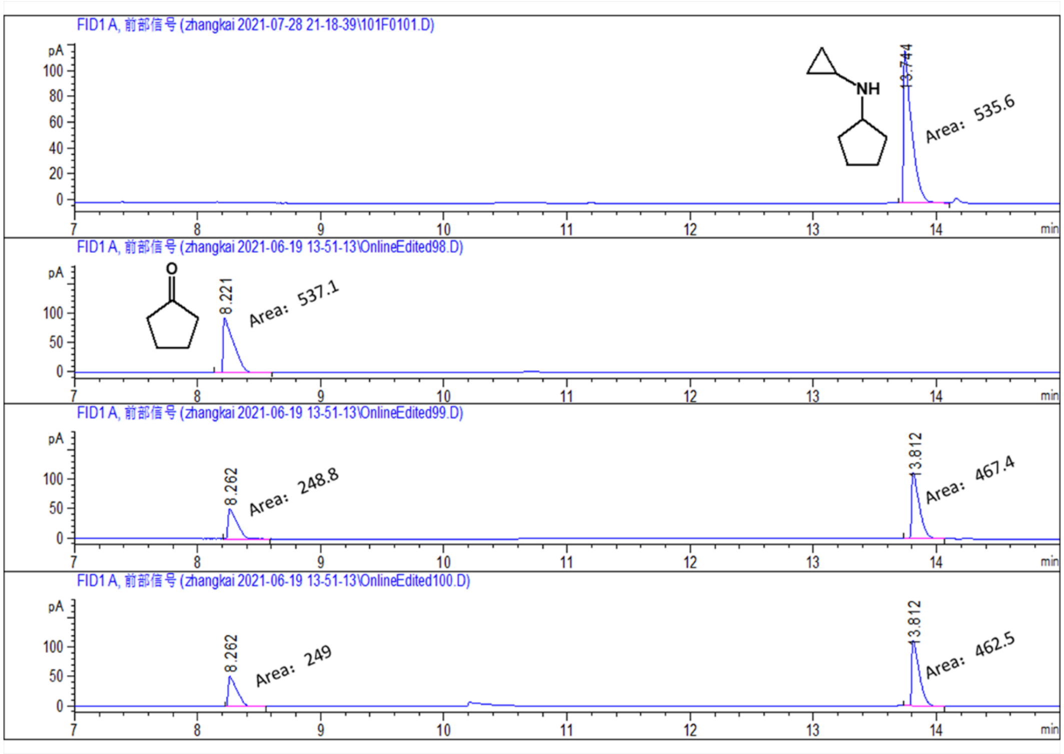

Figure S19. GC analysis: RedAm-catalyzing reductive amination of cyclopentanone **3** with cyclopropylamine **f** (1:1 molar ratio) showing standards of amine product **3f** (top), ketone **3** (second), biotransformation trace with *Ac*RedAm (third) and biotransformation trace with *Ba*RedAm (bottom).

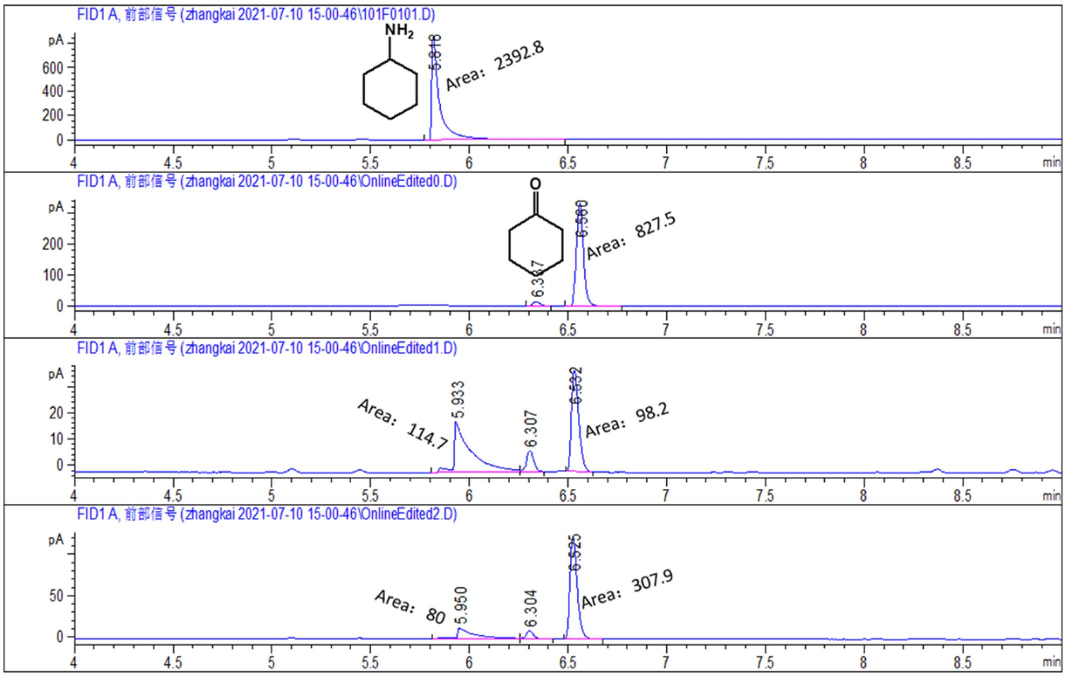

Figure S20. GC analysis: RedAm-catalyzing reductive amination of cyclohexanone **4** with NH_4Cl a (1:8 molar ratio) showing standards of amine product **4a** (top), ketone **4** (second), biotransformation trace with *Ac*RedAm (third) and biotransformation trace with *Ba*RedAm (bottom).

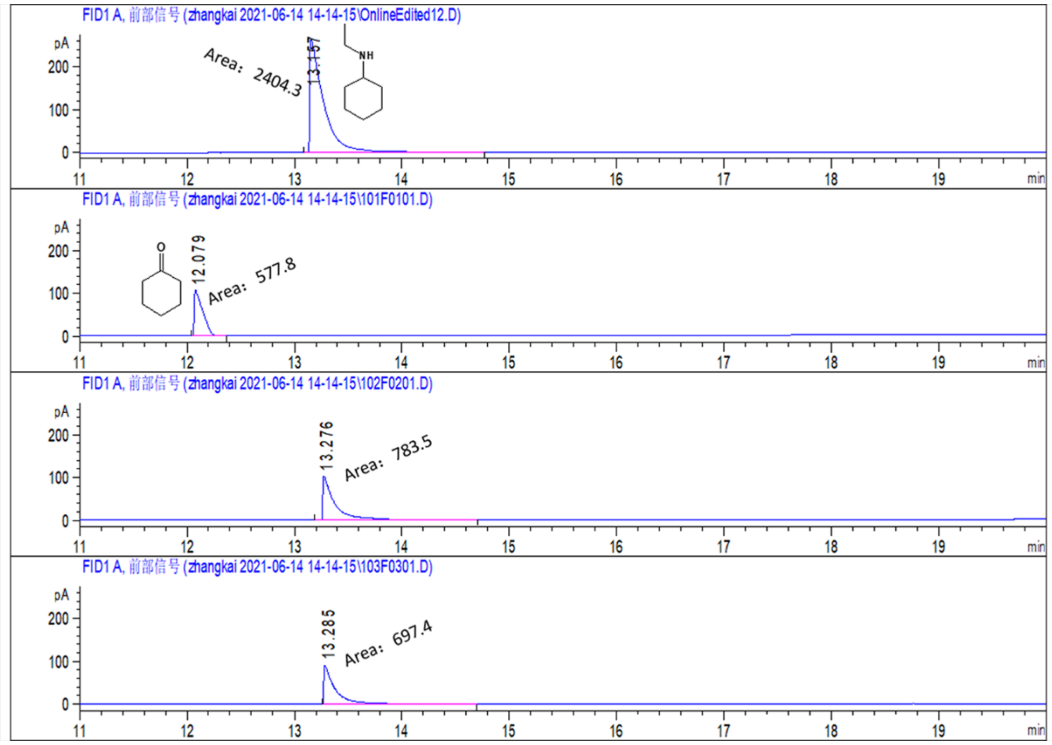

Figure S21. GC analysis: RedAm-catalyzing reductive amination of cyclohexanone **4** with ethylamine **b** (1:4 molar ratio) showing standards of amine product **4b** (top), ketone **4** (second), biotransformation trace with *Ac*RedAm (third) and biotransformation trace with *Ba*RedAm (bottom).

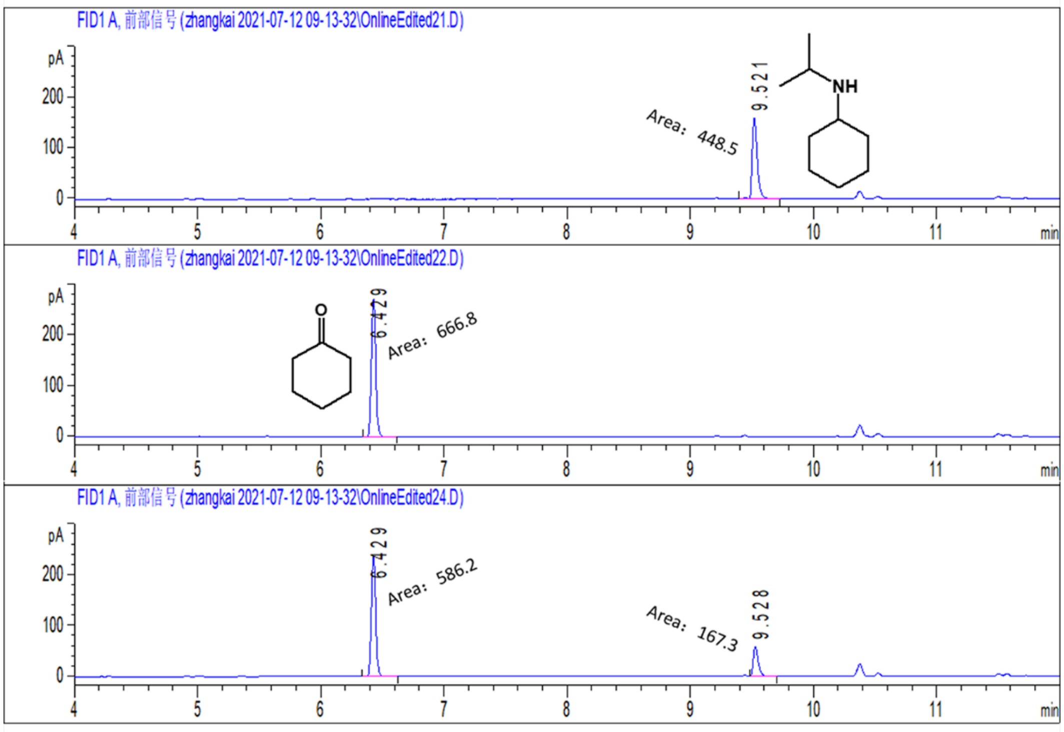

Figure S22. GC analysis: RedAm-catalyzing reductive amination of cyclohexanone **4** with isopropylamine **c** (1:20 molar ratio) showing standards of amine product **4c** (top), ketone **4** (second), and biotransformation trace with *Ba*RedAm (bottom).

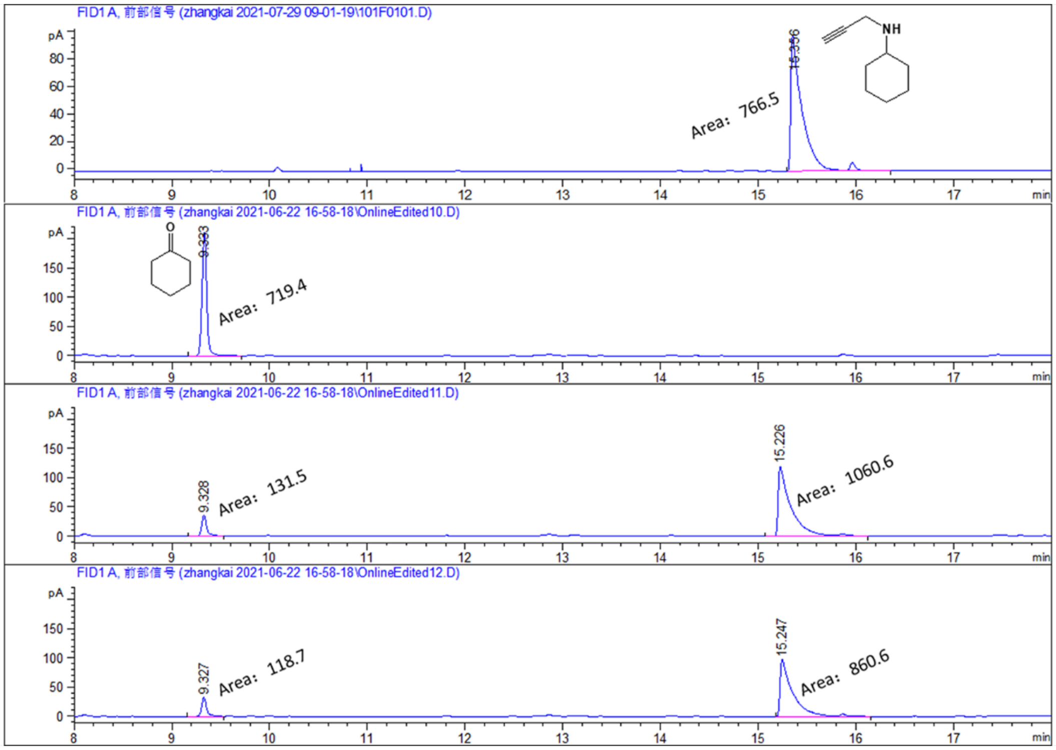

Figure S23. GC analysis: RedAm-catalyzing reductive amination of cyclohexanone **4** with propargylamine **e** (1:1 molar ratio) showing standards of amine product **4e** (top), ketone **4** (second), biotransformation trace with *Ac*RedAm (third) and biotransformation trace with *Ba*RedAm (bottom).

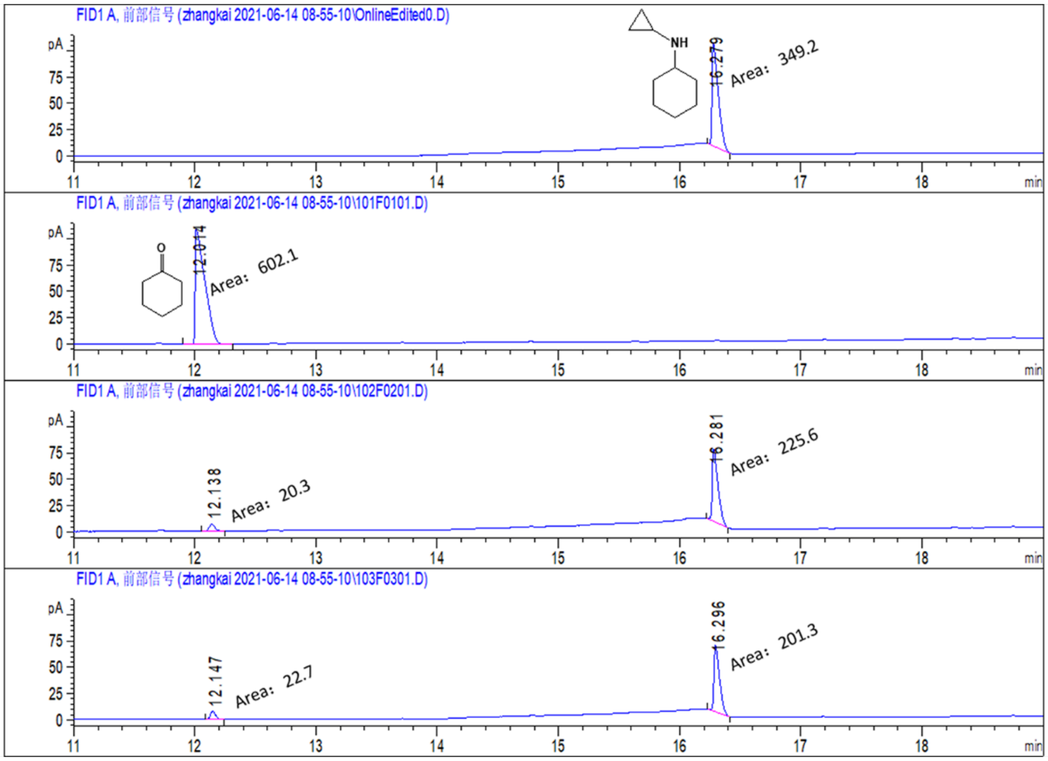

Figure S24. GC analysis: RedAm-catalyzing reductive amination of cyclohexanone **4** with cyclopropylamine **f** (1:1 molar ratio) showing standards of amine product **4f** (top), ketone **4** (second), biotransformation trace with *Ac*RedAm (third) and biotransformation trace with *Ba*RedAm (bottom).

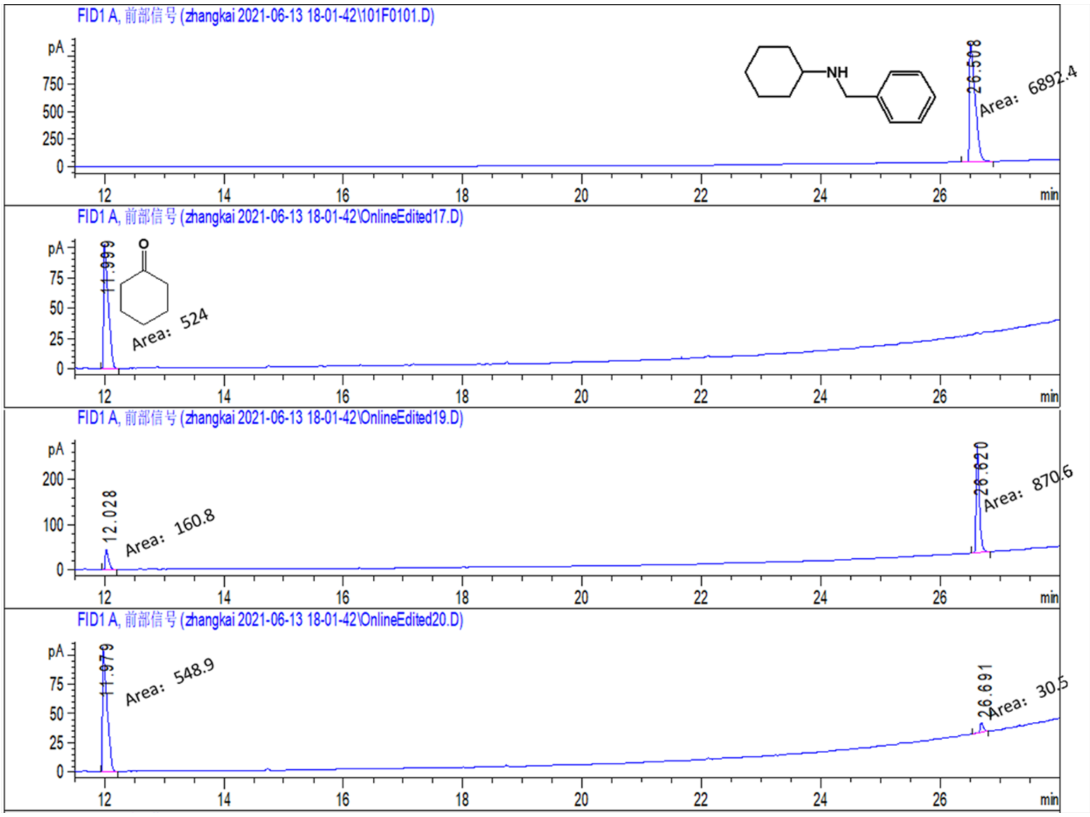

Figure S25. GC analysis: RedAm-catalyzing reductive amination of cyclohexanone **4** with benzylamine **i** (1:1 molar ratio) showing standards of amine product **4i** (top), ketone **4** (second), biotransformation trace with *Ac*RedAm (third) and biotransformation trace with *Ba*RedAm (bottom).

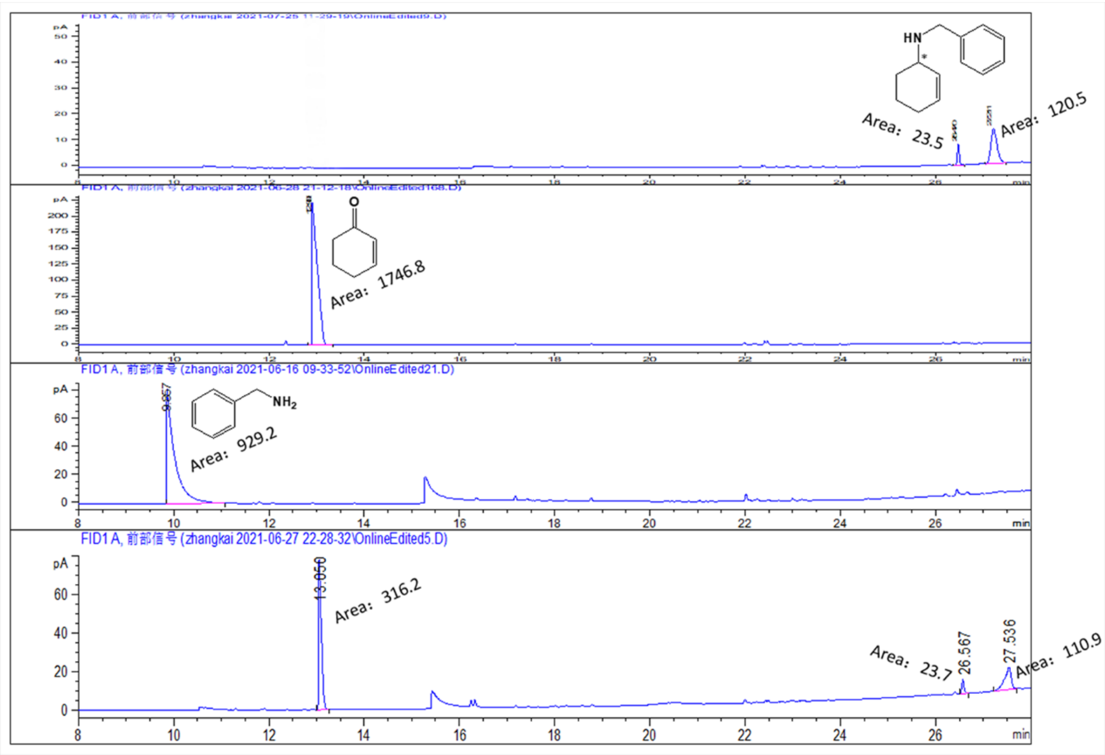

Figure S26. GC analysis: RedAm-catalyzing reductive amination of cyclohexenone **5** with benzylamine **i** (1:1 molar ratio) showing standards of amine product **5i** (top), ketone **5** (second), benzylamine **i** (third), and biotransformation trace with *Ac*RedAm (bottom).

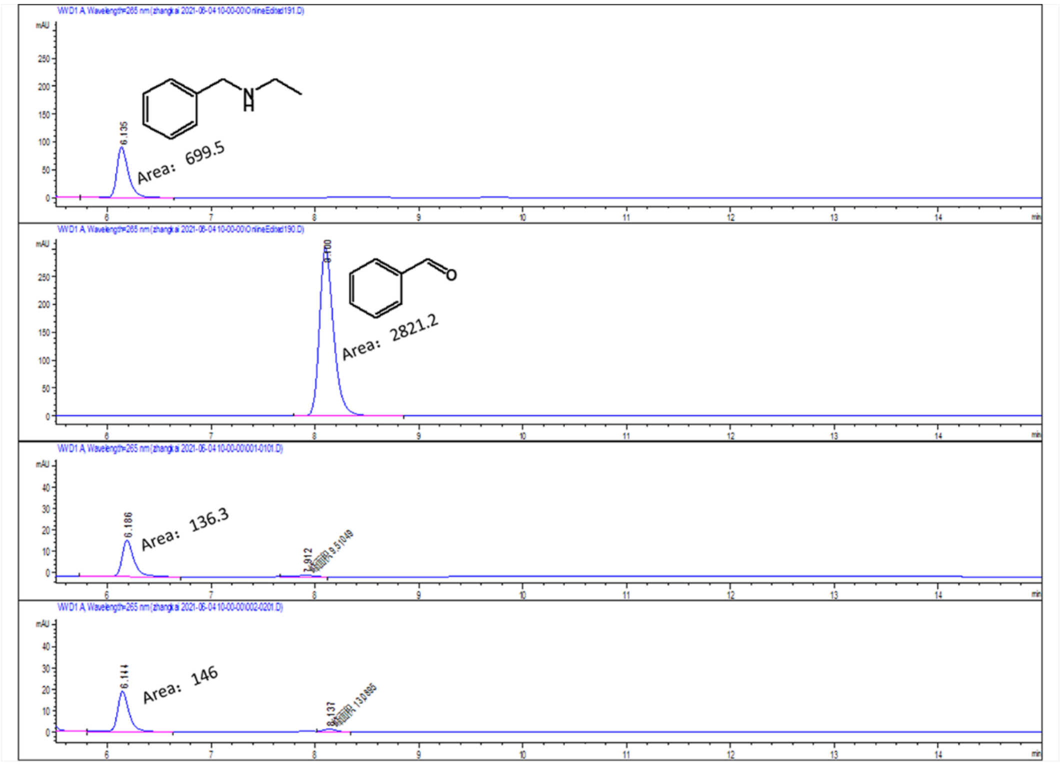

Figure S27. HPLC analysis: RedAm-catalyzing reductive amination of benzaldehyde **6** with ethylamine **b** (1:4 molar ratio), showing standards of amine product **6b** (top), benzaldehyde **6** (second), biotransformation trace with *Ac*RedAm (third) and biotransformation trace with *Ba*RedAm(bottom).

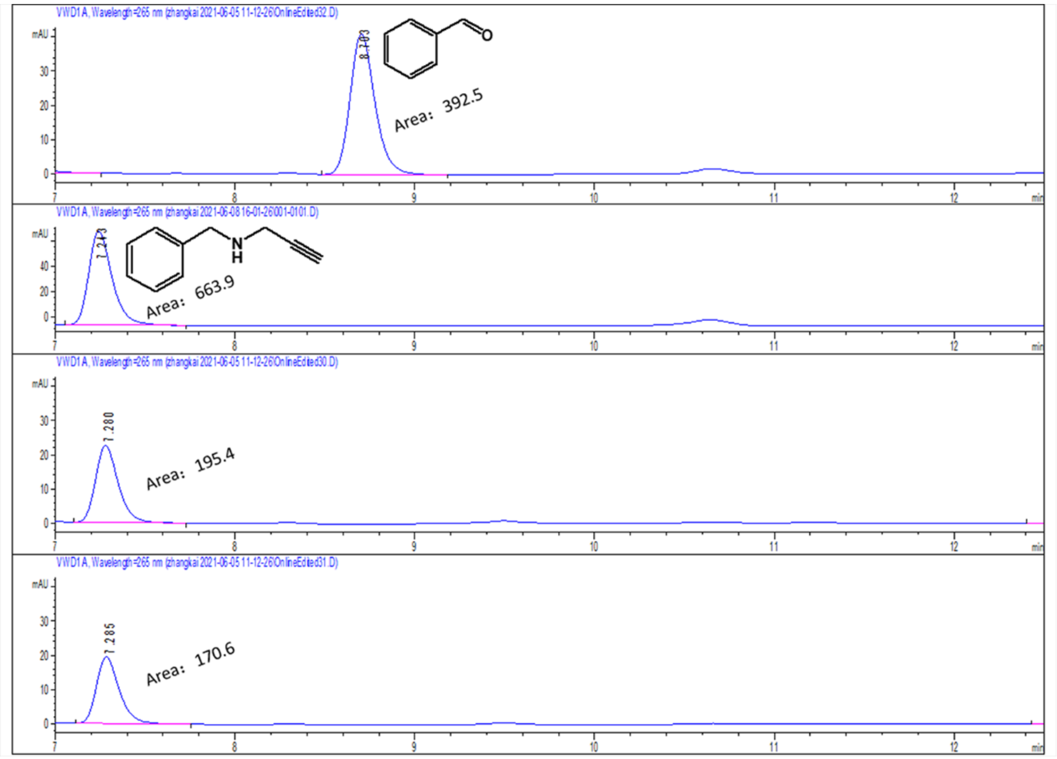

Figure S28. HPLC analysis: RedAm-catalyzing reductive amination of benzaldehyde **6** with propargylamine **e** (1:4 molar ratio), showing standards of benzaldehyde **6** (top), amine product **6e** (second), biotransformation trace with *Ac*RedAm (third) and biotransformation trace with *Ba*RedAm(bottom).

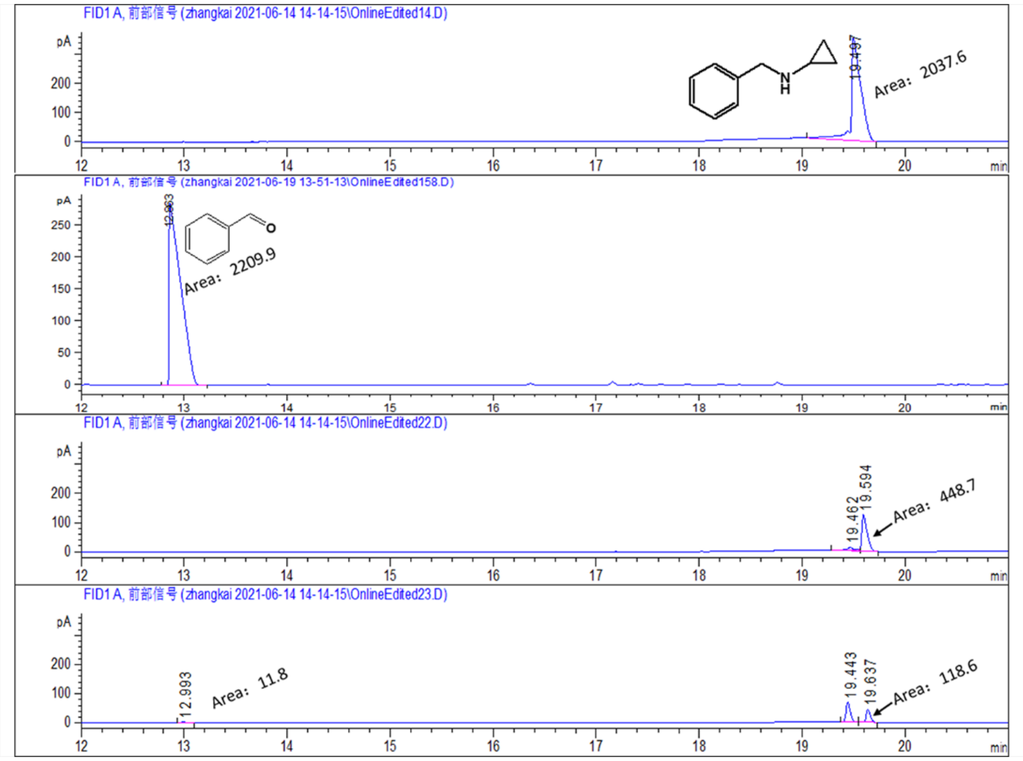

Figure S29. GC analysis: RedAm-catalyzing reductive amination of benzaldehyde **6** with cyclopropylamine **f** (1:4 molar ratio), showing standards of amine product **6f** (top), benzaldehyde **6** (second), biotransformation trace with *Ac*RedAm (third) and biotransformation trace with *Ba*RedAm(bottom).

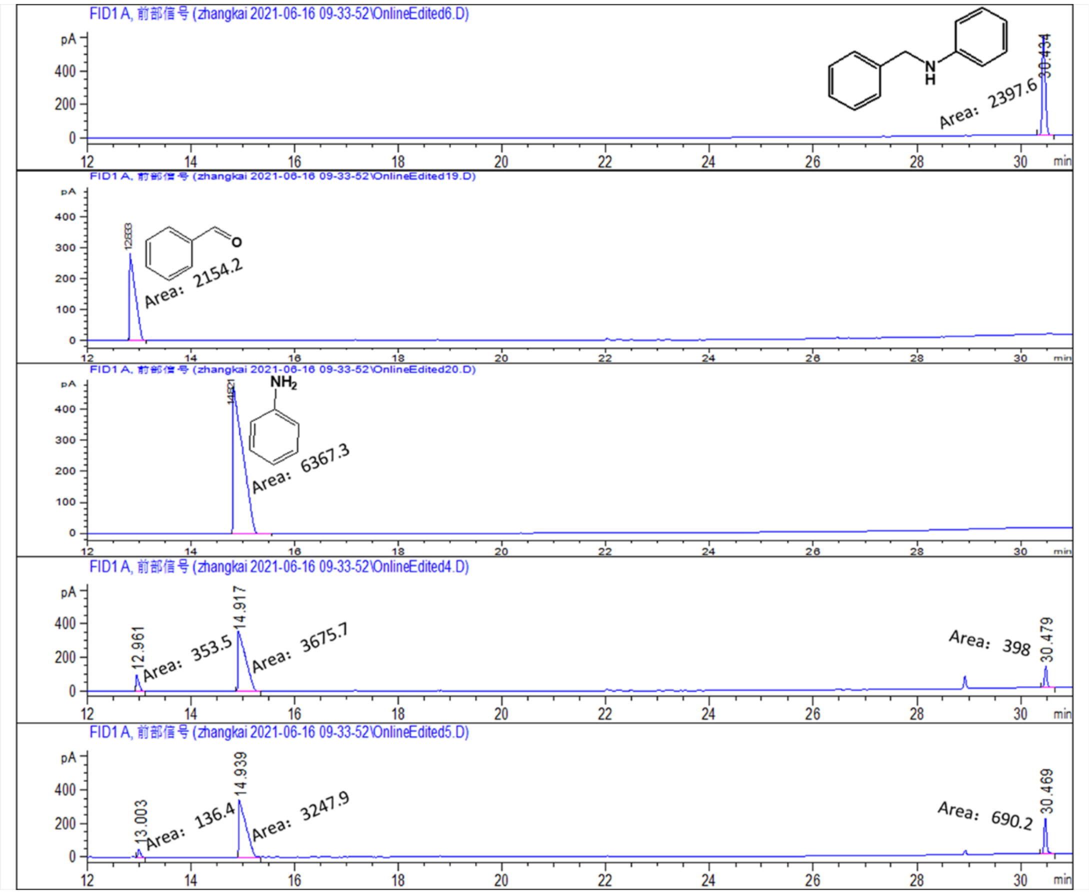

Figure S30. GC analysis: RedAm-catalyzing reductive amination of benzaldehyde **6** with benzeneamine **h** (1:4 molar ratio), showing standards of amine product **6h** (top), benzaldehyde **6** (second), benzeneamine **h** (third), biotransformation trace with *Ac*RedAm (fourth) and biotransformation trace with *Ba*RedAm(bottom).

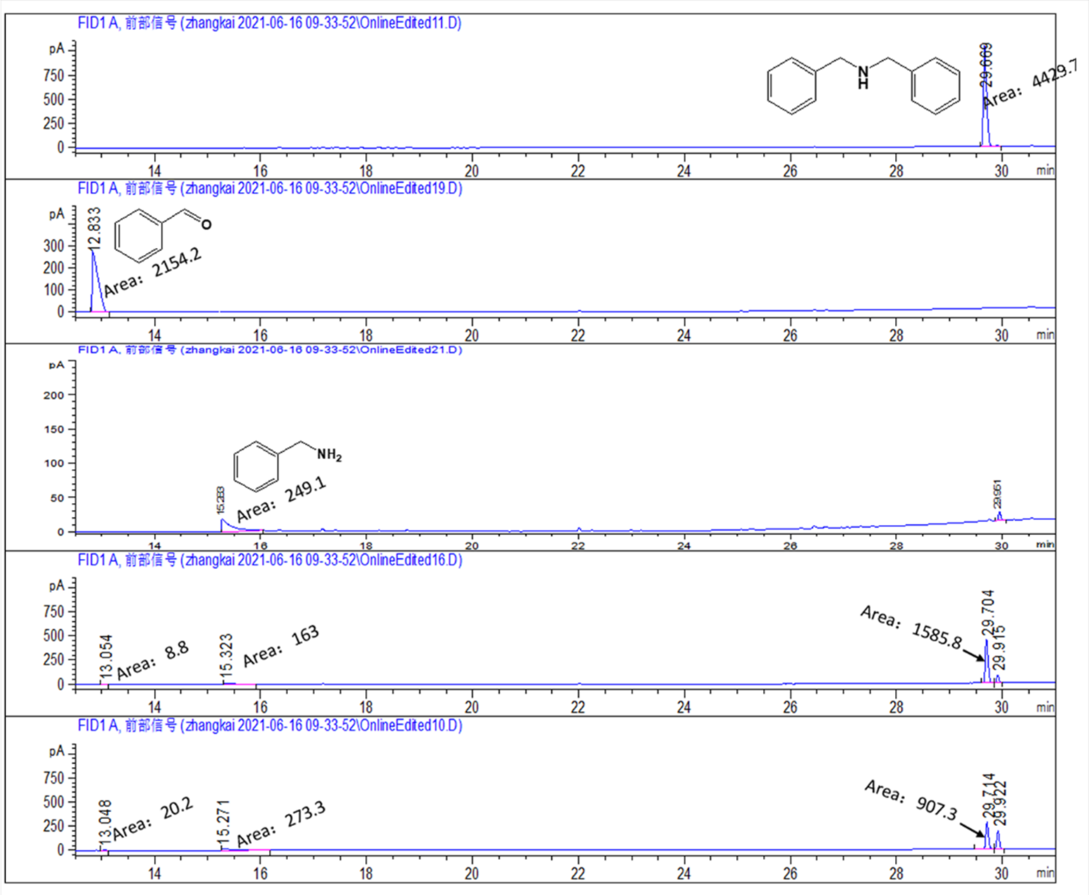

Figure S31. GC analysis: RedAm-catalyzing reductive amination of benzaldehyde **6** with benzylamine **i** (1:4 molar ratio), showing standards of amine product **6i** (top), benzalhyde **6** (second) and benzylamine **i** (third), biotransformation trace with *Ac*RedAm (fourth) and biotransformation trace with *Ba*RedAm(bottom).

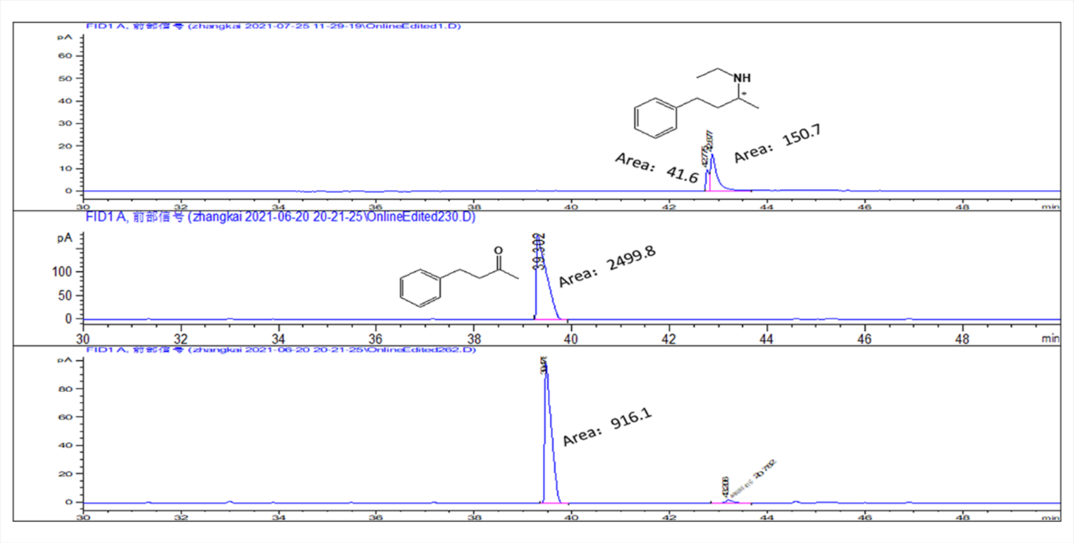

Figure S32. GC analysis: RedAm-catalyzing reductive amination of benzylacetone **7** with ethylamine **b** (1:20 molar ratio), showing standards of amine product **7b** (top), benzylacetone **7** (second), and biotransformation trace with *Ba*RedAm(bottom).

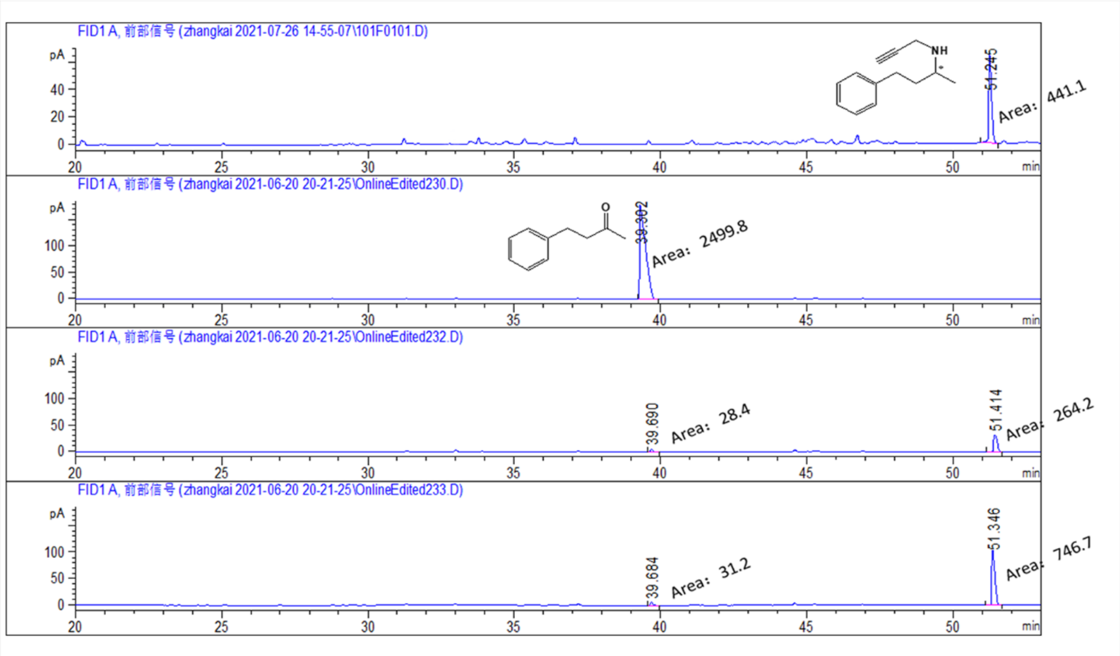

Figure S33. GC analysis: RedAm-catalyzing reductive amination of benzylacetone **7** with propargylamine **e** (1:20 molar ratio), showing amine product **7e** (top), standards of benzylacetone **7** (second), biotransformation trace with *Ac*RedAm (third) and biotransformation trace with *Ba*RedAm(bottom).

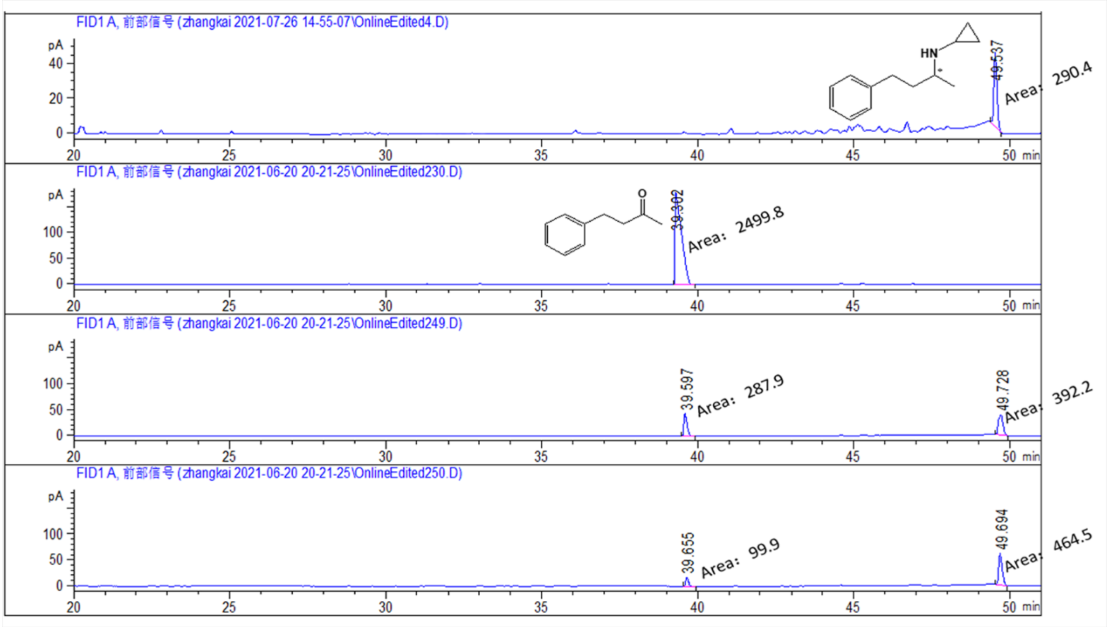

Figure S34. GC analysis: RedAm-catalyzing reductive amination of benzylacetone **7** with cyclopropylamine **f** (1:20 molar ratio), showing amine product **7f** (top), standards of benzylacetone **7** (second), biotransformation trace with *Ac*RedAm (third) and biotransformation trace with *Ba*RedAm(bottom).

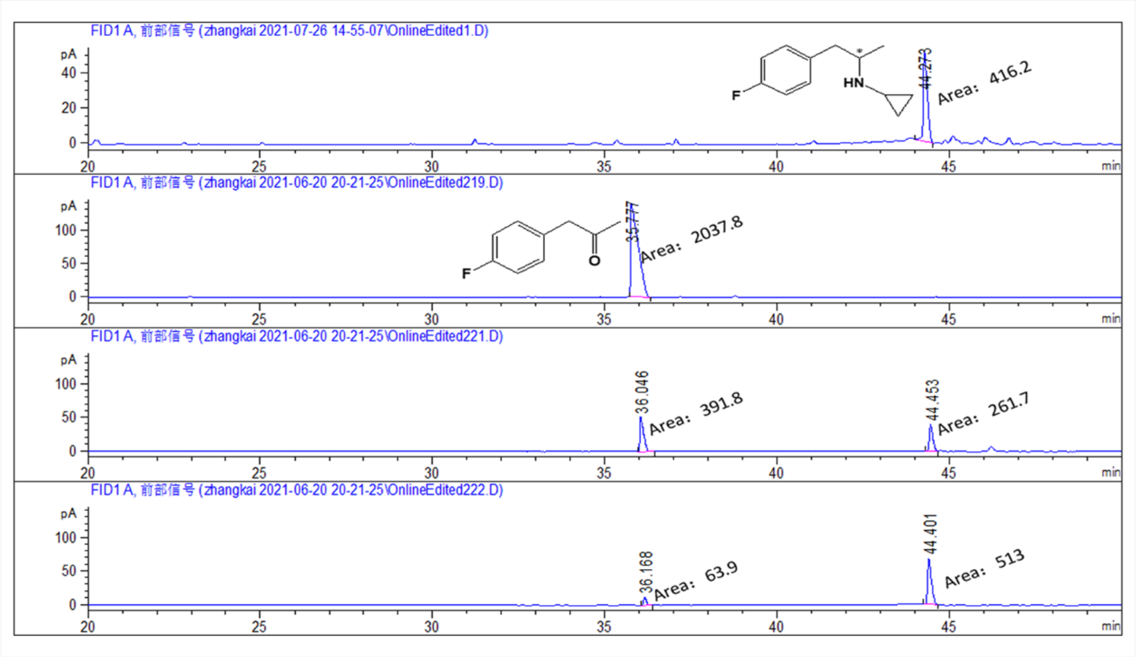

Figure S35. GC analysis: RedAm-catalyzing reductive amination of 4-F-phenyl acetone **8** with cyclopropylamine **f** (1:20 molar ratio), showing amine product **8f** (top), standards of 4-F-phenyl acetone **8** (second), biotransformation trace with *Ac*RedAm (third) and biotransformation trace with *Ba*RedAm(bottom)

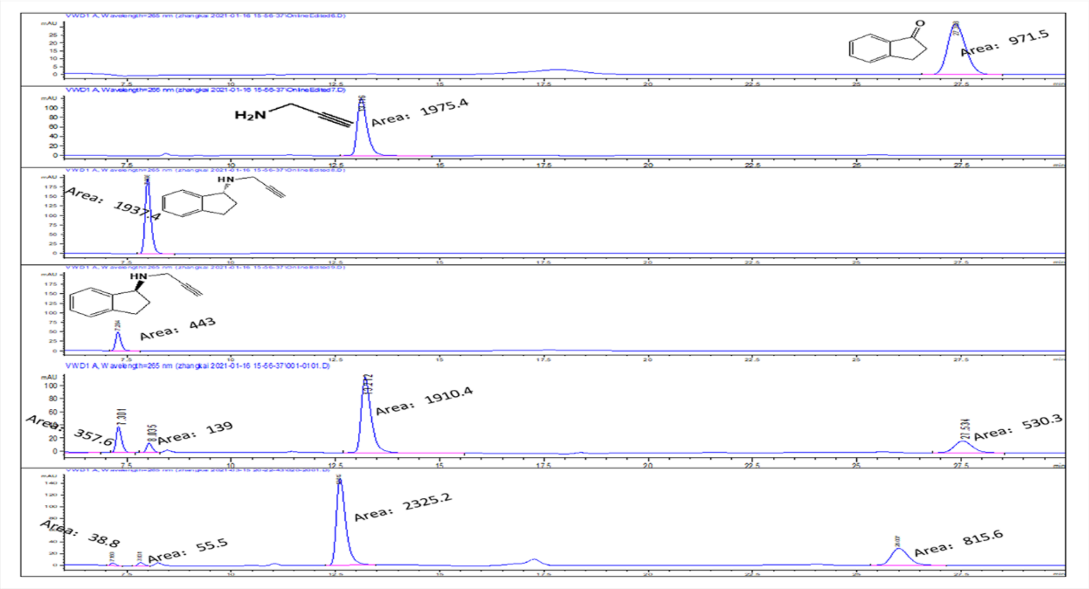

Figure S36. HPLC analysis: RedAm-catalyzing reductive amination of 1-indanone **9** with propargylamine **e** (1:50 molar ratio), showing standards of ketone **9** (top), propargylamine **e** (second), (*R*)**-9e** (third), (*S*)**-9e** (fourth), biotransformation trace with *Ac*RedAm (fifth) and biotransformation trace with *Ba*RedAm(bottom).

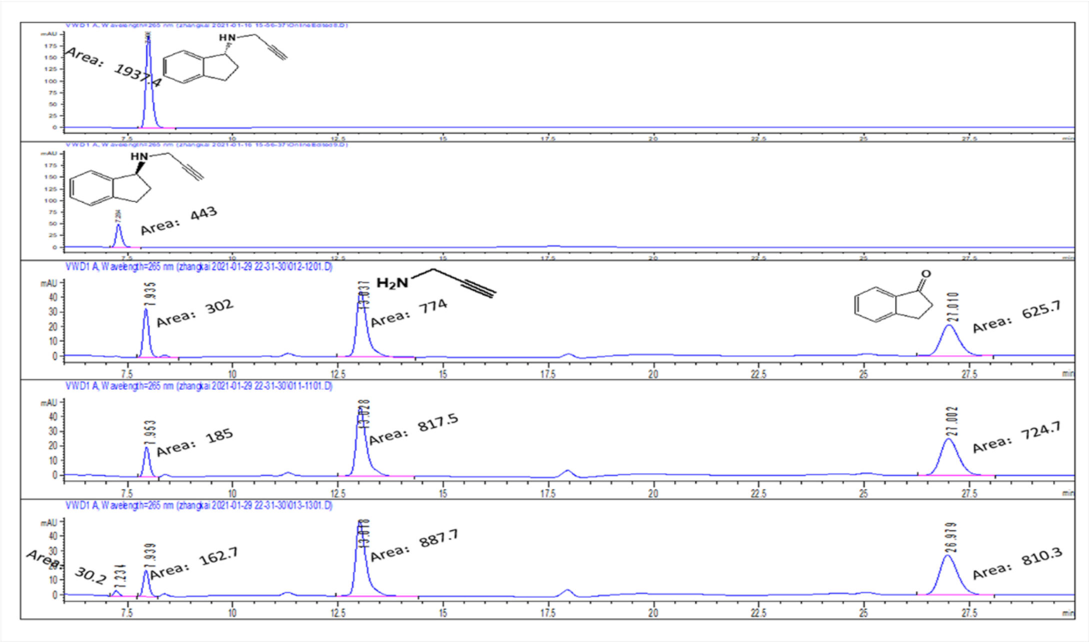

Figure S37. HPLC analysis: RedAm-catalyzing reductive amination of 1-indanone **9** with propargylamine **e** (1:50 molar ratio), showing standards of (*R*)-**9e** (top), (*S*)-**9e** (second), biotransformation trace with mutant Q237A (third), biotransformation trace with mutant Q237G (fourth), and biotransformation trace with mutant Q237S (bottom).

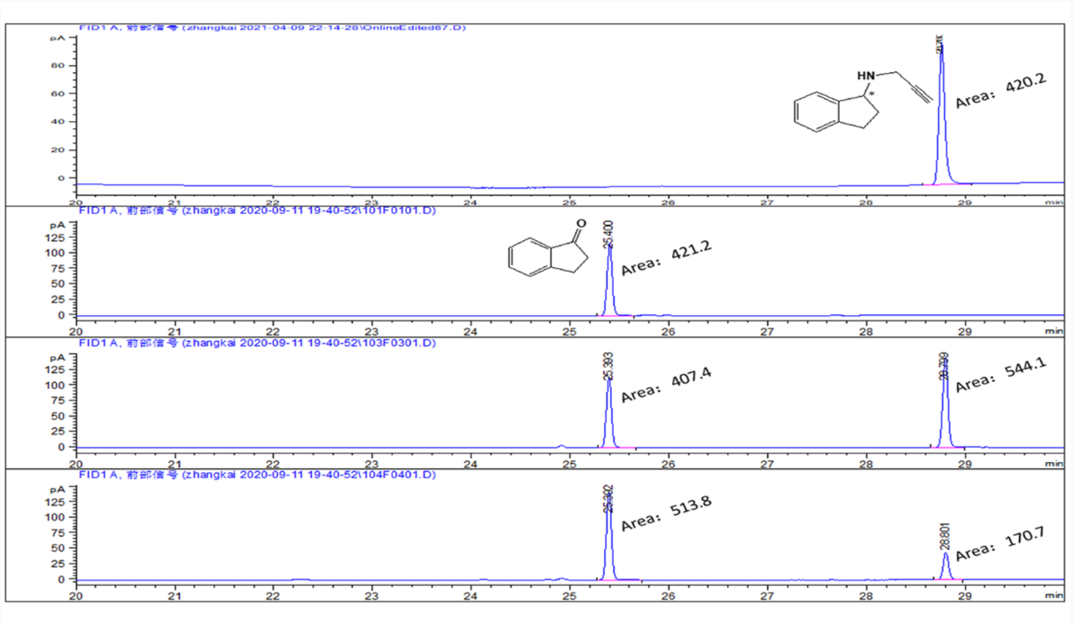

Figure S38. GC analysis: RedAm-catalyzing reductive amination of 1-indanone **9** with propargylamine **e** (1:50 molar ratio), showing standards of amine product **9e** (top), ketone **9** (second), biotransformation trace with *Ac*RedAm (third) and biotransformation trace with *Ba*RedAm(bottom).

Section 10. Codon-optimised sequences of candidate RedAms for expression in *E. coli*.

*As*RedAm

ATGAGCCGTAGCATCAGCATTCTGGGTCTGGGTGCGATGGGTACCGCGCTGGCGAGCCA
GTTCCTGGCGCAAAACTACAAGACCATCGTTTGGAACCGTAGCCCGGCGAAAGCGGTTT
GCCTGGCGGAGCAGGGTGCGCGTGCGGCGAGCACCGTTCTGGAAGCGCTGGCGGCGAG
CGACATGATCATTATCTGCCTGCTGGATAACAAGGCGGTGGAGGAAACCCTGACCCCGA
GCTTTGCGAGCCTGAGCGGCAAGACCGGCAAAACCATTATCATTAACCTGACCAACGGT
ACCCCGAACCAAGCGCGTAAACTGAGCACCCTGATCACCGACCAGGGCGCGCAATACAT
CCACGGTGGCATTATGGCGGTGCCGGTTATGGTGGGTACCCCGCACGCGATCCTGCTGT
ATAGCGGCGCGAGCGAGGAAATTTTCCGTCCGCTGGAGAGCGATCTGAGCCACCTGGGT
GTGGTTAAGTACTTTGGTCCGGATGCGGGTAGCGCGAGCCTGCACGATCTGGCGCTGCT
GAGCGGCATGTATGGCCTGTTCAGCGGTTTCTTTCACGCGACCGCGCTGGCGAAGAGCC
AGGGTACCGCGGCGACCGACTTTATGAGCATCCTGACCCCGTGGCTGACCGCGATGACC
CAGTATCTGGGTGTTCTGGCGAAACAAATTGACGATGAGGATTTCGCGACCCGTGGCAG
CAACCTGGAAATGCAACTGGCGGCGGTTCCGAACATCCTGACCGCGGGTGATGATCAGG
GCGTGAGCAGCGCGATGATCCTGCCGATTGTTCAACTGCTGGAAAAGGCGGTGCGTGAC
GGTTACGGTGGCGACGATCTGAGCCGTCTGATTGAGTATTTTAAACTGGAATAA

*Ath*RedAm

ATGTCAATTTCAATTTTTGGCTTAGGCGCCATGGGCACCGCACTGGCCTCTCGCTTTCTG
GAAGAAAAATATAAAGTGACCATTTGGAATCGTAGTCTGGAAAAAGCCTCTCCGCTGCT

GAAAAAAGGTGCCACCTTAAGTCATACCGCCGTGGATGGCATTCATGCCTCTAATTTAA
TTATTATTTATCTGCTGAATAATGCAGCCGTTCAGGCATCACTGGATAGTACCTTAAATC
AGTTACAGGGTAAAACCATTATTAATCTGACCAATCGTACCCCGGAACAGGCCCAGACC
CTGTCAGATCTGATTATGAATCATAGCGCCCAGTATATTCATGGTCGCATTATGGCAACC
CCGTCAATGATTTCTTCTCCGCATGCCCTGATTCTGTATAGCTCTAGTAGCGGTGCCTTTA
AAGCAAGTAAAGCAGATCTGAGCGTGTTAGCCAAATGTCTGTTTCTGGGCGATGGTGCC
GGTAGTGCAAGTTTACATGATCTGGCACTGCTGAGTGGTATGTATGGCCTGTTTAGCGGC
TTTTTACATGCCACCGCCCTGGTTCGCTCTTCAACCCCGGCCGTGAAATTTGTGGATCTG
TTAGTTCCGTGGTTAGGCGCAATGACCGAATATACCAAAGGTATGGCCAAACAGATTGA
TGATGGTAAATATGCAAGCGAAGGTTCTAATCTGTCTATGCAGTTAGCAGCCATTCAGA
ATATTATTGATGCATCAGCCGCACAGCAGGTGAGCGCAAATTTTATTCGTCCGATGAAA
GAATTTATGCAGAAAGCAGTTGCAGCCGGTCGCGGCGGCGATAATATTAGCTCACTGAT
TGATTTTGTGAAACCGACCTAA

*Ac*RedAm

ATGAGCACCATTACCCTGTTCGGTCTGGGTGCGATGGGCAAGGCGCTGGCGGCGAAGTA
CATCGAGAAAGGCTATACCACCACCATTTGGAACCGTACCCCGAGCAAAGCGGCGCCGC
TGGTTGAGAAGGGTGCGAAACTGGCGAACACCGTTGGTGAAGGTCTGGCGAGCGCGGA
CCTGATCATTCTGTGCCTGCTGGATAACGCGAGCGTGCGTCAAACCCTGGACCAAGCGA
CCGCGGCGCTGAACGGCAAGACCGTTATCAACCTGACCAACGGTACCCCGAGCCAGGCG
CGTGAGACCAGCGAATGGGTGATTAGCCACGGCGCGCAATACATCCACGGTGGCATTAT
GGCGGTGCCGGATATGATCGGTAGCCCGCACGCGGTTCTGCTGTATAGCGGCGAGAGCG
CGGAAACCTTCAGCCGTGTTGAAGCGCACCTGAGCCACCTGGGTACCAGCAAATTTCTG
GGTACCGACCCGGGTAGCGCGAGCCTGCACGATCTGGCGCTGCTGAGCGGCATGTACGG
CCTGTTCAGCGGCTTCTTTCATGCGACCGCGCTGGTTAAAAGCCAACCGGGTACCACCGC
GACCGGTTTTGTTCAACTGCTGACCCCGTGGCTGAGCGCGATGACCCACTACCTGGGTGC
GCTGGCGAAACAGATTGACGAGGGTGATTATGCGACCCAAGGCAGCAACATGGCGATG
CAGGTGACCGGTGTTCAAAACATCGTTCGTGCGAGCGAGGAAGCGGGCGTTACCGCGGA
CCTGATCATGCCGATTCTGGGTCGTATGACCCGTGCGGCGGAAGCGGGTTATGCGGACG
TGGATGTTAGCGCGGTGATCGAGTTTATGAAGGAATAA

*Ba*RedAm

ATGCGTGAACCGATCGTGAGCGCGCATACCGAACGCGCCGTTGAAAGCCGTGGTGCCGA
TCGCGGTAGTGCGGTGACCGTTATTGGTCTGGGCAGTATGGGTAGTGCCCTCGCGGGTG
CCGTTCTGGAAGCCGGCTATCCAACGACCGTTTGGAATCGTACGGCCGGTAAAGCCGAG
CCGCTGGTTCGTCGCGGCGCCGCCCGTGCCGCGACGGTTGCCGAAGCGGTTAGTGCGAG
CCCGACGGTGATCGCGTGCGTTCTGGATTATCGCGCGCTGCGCGAGATTCTGAGTACCG
CCGGTGATGCGCTGGCCGGTCGTACGGTTGTGAATCTCACGAACGGCACGCCAACCGAA
GCGCGTGAAACGGCGGCGTGGGTTGAAGGCCATGGTGCCCGTTATCTGGATGGCGGTAT
CATGGCGGTGCCGGAAATGATTGGCGGCGCGGAAAGCCTCGTGCTCTACAGCGGCAGCG
CCGAAGCGTTCGAAACCGTTGAACCGGTTCTGCGCCGCTTCGGCAGTGCGATGTATCTG
GGTGCGGATCCGGGTCTGGCCAGTCTGCACGATCTGGCGCTGCTGGCGGGCATGTATGG
TCTGTTCGCCGGCTTTCTGCATGCGGTTGCGCTGGTTGGTACCGAGGGTGTTCGTGCCAC
CGAATTTACCAGCAGTCTGCTCATCCCGTGGCTGCAAGCCATGACCGCCACGCTGCCAG
AAGCGGCGGCGCAAATTGATGCCGGTGATTACGCCGCCACCGGTAGTCGTCTGGATATG
CAAGCCGTTGCGCTGGCGAACATCGTGGAAGCCAGTCGCAGCCAAGGCATTCGCCCGGA

TCTGATGCTCCCAATCCAAGCCCTCGTGGAACGTCGCGTGGCCAAAGGTGGCGGTGGCG
AAGATATCGCCGCGGTTGTTGAAGAAGTGCGCGGTTAA
